# Starter-Culture-Dependent Effects of *Lactiplantibacillus plantarum* and *Lactobacillus delbrueckii* subsp. *bulgaricus* Fermentation on Nutritional Quality, Flavor Characteristics and Metabolite Profiles of *Flammulina velutipes* Roots

**DOI:** 10.3390/foods15172973

**Published:** 2026-08-24

**Authors:** Haixu Zhou, Meng Zhang, Minghao Chen, Shuhui Zhang, Yang Wu, Haijuan Nan, Zhipeng Qu

**Affiliations:** School of Food Science, Henan Institute of Science and Technology, Xinxiang 453003, China

**Keywords:** *Flammulina velutipes* roots, lactic acid bacteria, flavor, untargeted metabolomics, starter-culture-dependent

## Abstract

*Flammulina velutipes* roots, abundant edible mushroom by-products, have potential for value-added utilization. This study evaluated the effects of *Lactiplantibacillus plantarum* (*Lpb. plantarum*) and *Lactobacillus delbrueckii* subsp. *bulgaricus* (*Lab. bulgaricus*) fermentation on the physicochemical properties, antioxidant activity, flavor characteristics, sensory characteristics, and metabolite profiles of *F. velutipes* roots. Fermentation significantly increased soluble dietary fiber (SDF) from 1.537 to 1.722 g/100 g and DPPH radical scavenging activity from 27.88% to 59.92% in *Lpb. plantarum*-treated samples. GC-IMS analysis showed that fermentation increased several flavor-active aldehydes, esters, alcohols, organic acids, and ketones, thereby improving aroma complexity. LC-MS-based untargeted metabolomics revealed that amino acid metabolism, fatty acid metabolism, organic acid metabolism, and phenylpropanoid-related pathways were closely associated with flavor formation and antioxidant enhancement. Both *Lpb. Plantarum* and *Lab. bulgaricus* fermentation enhanced the accumulation of organic acids, sugar alcohols, and aromatic precursors, supporting improved antioxidant activity. Notably, *Lpb. plantarum* elicited broader metabolic shifts involving hydroxy fatty acids, esters, while *Lab. bulgaricus* more strongly promoted organic acid and aromatic metabolite accumulation, contributing to a more acidic and malty flavor profile. Sensory evaluation and electronic tongue analysis further confirmed that fermentation enhanced aroma and palatability, while increasing sourness and reducing bitterness and astringency. These results suggest that starter-culture-dependent lactic acid bacteria fermentation is an effective strategy for improving the functional and sensory characteristics of *F. velutipes* root by-products.

## 1. Introduction

*Flammulina velutipes*, also known as *F. filiformis* in East Asia, is one of the most widely cultivated edible mushrooms worldwide [1]. The industrial processing of *F. velutipes* inevitably generates substantial root waste, accounting for approximately 10–15% of the fruiting body biomass. In 2023, *F. velutipes* production reached 1.87 million tons according to the China Edible Fungi Association (https://bigdata.cefa.org.cn/) (accessed on 15 February 2025), meaning that approximately 187,000 tons of *Flammulina velutipes* roots-the basal stipe fragments-were discarded. These roots are rich in proteins, polysaccharides, dietary fiber, phenolics, and flavonoids and exhibit notable antibacterial and antioxidant activities [2,3]. However, they are largely discarded as industrial waste, resulting in both resource loss and environmental burden. Therefore, the development of effective strategies for the valorization of *F. velutipes* roots is of considerable interest.

Lactic acid bacteria (LAB) fermentation is an effective bioprocessing strategy to improve the nutritional value, bioactivity, and sensory characteristics of agricultural by-products [4]. Among LAB species, *Lactiplantibacillus plantarum* (*Lpb. plantarum*) and *Lactobacillus delbrueckii* subsp. *bulgaricus* (*Lab. bulgaricus*) possess distinct metabolic characteristics. *Lpb. plantarum* exhibits strong polysaccharide-degrading capacity and can generate diverse flavor-active metabolites, whereas *Lab. bulgaricus* is characterized by rapid acidification and aroma formation [5,6]. These complementary metabolic properties make them suitable agents for restructuring the fibrous matrix of *F. velutipes* roots and improving their functional and sensory characteristics.

Flavor is a key factor driving consumer acceptance of fermented foods. With its high sensitivity, rapid detection, simple sample preparation, and intuitive data visualization, gas chromatography-ion mobility spectrometry (GC-IMS) has become a powerful tool for volatile compound analysis [7]. To further relate instrumental measurements to consumer perception, sensory evaluation, and electronic tongue (E-tongue) analysis can be integrated to characterize both aroma and taste attributes, providing a comprehensive assessment of flavor [8,9]. However, flavor changes represent only the phenotypic outcome of complex metabolic reprogramming during fermentation. Untargeted metabolomics based on liquid chromatography-mass spectrometry (LC-MS) enables comprehensive profiling of small-molecule metabolites. It has become an important tool for elucidating fermentation-induced alterations [10]. Previous studies have successfully applied LC-MS metabolomics to reveal biochemical changes in various fermented food matrices, which offers mechanistic insights into quality improvement during fermentation [11].

Although *F. velutipes* roots have been investigated for polysaccharide extraction and dietary supplementation [3,12,13,14], research on their biotransformation through LAB fermentation remains limited. Moreover, existing research on fermented edible-fungus by-products has mainly focused on either physicochemical characteristics or volatile flavor profiles, with little attention given to linking sensory changes with global metabolic responses [15,16,17,18]. Consequently, the mechanisms by which different LAB strains regulate nutritional components, flavor profiles, and antioxidant activity in *F. velutipes* roots remain largely unclear.

Therefore, this study examined the starter-culture-dependent effects of *Lpb. plantarum* and *Lab. bulgaricus* fermentations on *F. velutipes* roots. A comprehensive evaluation was conducted to characterize the fermentation-induced changes in physicochemical properties, antioxidant activity, sensory characteristics, taste attributes, volatile compounds, and non-volatile metabolites, using conventional analytical methods, sensory evaluation, E-tongue, GC-IMS, and LC-MS-based untargeted metabolomics. The objectives were to elucidate LAB-induced modifications in nutritional, functional, and sensory properties, and to pinpoint key flavor-associated metabolites. These findings support the valorization of *F. velutipes* root by-products through *Lpb. plantarum* and *Lab. bulgaricus* fermentations.

## 2. Materials and Methods

### 2.1. Materials and Reagents

*F. velutipes* roots were purchased from Xinxiang Xinghe Biotechnology Co., Ltd. (Xinxiang, China). *Lactiplantibacillus plantarum* (*Lpb. plantarum*) (GDMCC NO.1.2685) and *Lactobacillus delbrueckii* subsp. *bulgaricus* (*Lab. bulgaricus*) (ATCC 11842) were purchased from the Guangdong Microbial Culture Collection Center (Guangzhou, China) and Zhengzhou Hehe Bioengineering Technology Co., Ltd. (Zhengzhou, China), respectively. De Man–Rogosa–Sharpe (MRS) broth and agar medium (No. R42468), 2,2-diphenyl-1-picrylhydrazyl (DPPH) (No. R43058) and Folin–Ciocalteu reagent (No. S29851) were purchased from Tianjin Huasheng Chemical Reagent Co., Ltd. (Tianjin, China). MRS-selective medium (No. wp1003) was purchased from Shenzhen Weitong Biotechnology Co., Ltd. (Shenzhen, China). All other analytical-grade reagents were purchased from China National Pharmaceutical Group Corporation.

### 2.2. Preparation of Starter Culture

Freeze-dried *Lpb. plantarum* and *Lab. bulgaricus* cultures were rehydrated in sterile water, followed by activation on MRS agar at 37 °C for 48 h and subsequent propagation in MRS broth at 37 °C for another 48 h. Cells were collected by centrifugation (4000× *g*, 10 min, 4 °C), rinsed twice with sterile saline, and resuspended to approximately 2.2 × 10^9^ CFU/mL as confirmed by plate counting.

### 2.3. Fermentation of F. velutipes Roots

Fermentations were conducted separately for each strain to clearly attribute metabolic outcomes to their specific activities. Briefly, *F. velutipes* roots (2 × 2 × 2 cm, 100 g) were combined with distilled water (100 mL), supplemented with glucose (2%, *w*/*w*) and salt (1%, *w*/*w*) to promote initial LAB growth and suppress spoilage organisms [19]. The mixture was transferred into 250 mL bottles and sterilized at 115 °C for 10 min, a condition that preliminary tests confirmed that did not cause significant browning or textural collapse associated with severe nutrient loss. After cooling, activated *Lpb. plantarum* or *Lab. bulgaricus* cultures were inoculated at 3% (*w*/*w*, approximately 2.7 × 10^7^ CFU/mL). The fermentation process was carried out at 37 °C over a time course of 0, 8, 16, 24, 32, 40, and 48 h. At designated intervals, *F. velutipes* roots were harvested, lyophilized under vacuum, and pulverized for further analysis. Based on time-course changes in SDF, total sugar, pH, total polyphenols, total flavonoids, and antioxidant activity, samples fermented for 48 h were selected for GC-IMS, E-tongue, sensory evaluation, and metabolomics analyses [20]. Uninoculated 0-h samples served as the control for physicochemical measurements, whereas uninoculated samples incubated under the same conditions for 48 h served as the control (CK) for flavor and metabolomic assessments. All control samples were processed in parallel without starter addition.

### 2.4. Determination of pH and Titration of Acidity

The pH was measured using a pH meter (Shanghai Instrument and Electrical Instrument Co., Ltd., Shanghai, China), and titratable acidity was determined according to the method of Cardinali et al. [21].

### 2.5. Analysis of Dietary Fiber and Total Sugar

All chemical composition analyses were performed on freeze-dried and comminuted samples. The contents of soluble dietary fiber (SDF), insoluble dietary fiber (IDF), and total dietary fiber (TDF) were determined in line with GB 5009.88-2023 [22], whereas ash and protein contents were measured according to GB 5009.4-2016 and GB 5009.5-2016 [23,24], respectively. Equation (1) details the calculations of SDF, IDF, and TDF.

(1) $$X\;(g/100\;g)=\frac{{\bar{M}}_{residue}-M_{P}-M_{A}-M_{B}}{\bar{M}\times f}\times 100$$

where

*X* = content of dietary fiber in the test sample (g/100 g);
${\bar{M}}_{residue}$ = average mass of the duplicate sample residues and crucibles (g);
*M_P_* = mass of protein in the residue, in grams (g);
*M_A_* = mass of ash in the residue, in grams (g);
*M_B_* = mass of the reagent blank, determined separately according to the national standard method, in grams (g);
$\bar{M}$ = average sample mass of the duplicate portions, in grams (g);
*f* = correction factor (f = 1, if the sample has not undergone pretreatment procedures such as drying or defatting).

The phenol-sulfuric acid method with glucose as the standard was employed for total sugar determination [25]. The resulting calibration curve (y = 1.3312x − 0.0403, R^2^ = 0.9994) correlated absorbance (y) with glucose equivalent concentration (x, mg/mL) (Figure S1). Total sugar content (mg/g) was subsequently calculated using Equation (2).

(2) $$\mathrm{Total}\;\mathrm{sugar}\;\mathrm{content}\;(\mathrm{mg}/g)=\frac{C\;\times \;V\;\times \;D}{M}$$

where *C* (mg/mL) is the glucose equivalent concentration derived from the standard curve, *V* (mL) is the constant volume of the sample extraction, D is the dilution factor, and *M* (g) is the sample weight. Results were expressed as mg glucose equivalents per gram of dry sample (mg/g, as glucose equivalent).

### 2.6. Total Phenolic Content (TPC) and Total Flavonoid Content (TFC)

TPC was determined using the Folin–Ciocalteu method [26]. Briefly, 0.25 mL of diluted sample was mixed with 0.25 mL of Folin–Ciocalteu reagent (1:1, *v*/*v*). The mixture was supplemented with 0.5 mL of saturated sodium carbonate (200 g/L) and 4.0 mL of distilled water, incubated for 45 min at room temperature, and then measured for absorbance at 760 nm. The results were expressed as gallic acid equivalents (GAE).

TFC was determined using a colorimetric procedure [27]. In brief, 0.25 mL of extract was mixed with 0.75 mL methanol, 0.05 mL 10% (*w*/*v*) AlCl_3_, 0.05 mL 1 M potassium acetate, and 1.4 mL distilled water. After 30 min incubation at room temperature, the absorbance at 420 nm was recorded and calculated as rutin equivalents (RE).

### 2.7. Analysis of Antioxidant Activity

Antioxidant assays were performed on supernatants obtained by ultrasonication (30 min) of freeze-dried material in 70% methanol (1:20, *w*/*v*).

DPPH radical scavenging activity was conducted following the protocol of Baliyan et al. [28]. Briefly, 50 µL of extract was mixed with 3 mL of 60 µM DPPH solution and allowed to react in the dark for 30 min. The absorbance at 517 nm was recorded, and scavenging activity was calculated using Equation (3).

(3) $$DPPH\;scavenging\;activity\;(\%)=\frac{\left(A_{0}-A_{S}\right)}{A_{0}}\times 100$$

where *A_S_* is the sample absorbance and *A*_0_ is the absorbance of the control.

Reducing power was determined using a standard ferricyanide protocol as described by Li et al. [29]. In this assay, 1 mL of the sample was incubated with phosphate buffer (0.2 M, pH 6.6, 2.5 mL) and 1% K_3_[Fe(CN)_6_] (2.5 mL) at 50 °C for 20 min. The reaction was stopped by adding 2.5 mL of 10% trichloroacetic acid (TCA), followed by centrifugation at 1760× *g* for 15 min. An aliquot (2.5 mL) of the supernatant was then mixed with distilled water (2.5 mL) and 0.1% FeCl_3_ (0.5 mL) for 30 min at room temperature. The absorbance of the colored product was measured at 700 nm against a water blank; the resulting value (A700) was directly proportional to the reducing potential of the sample. To express the reducing power as mg Vc equivalent per gram of dry weight (DW), a calibration curve was constructed using ascorbic acid standards (0, 50, 100, 150, 200, and 250 µg/mL), following the same ferricyanide reduction protocol. The linear regression equation obtained was y = 0.00344 + 0.00184x (R^2^ = 0.997) (Figure S2), where x is the Vc concentration (µg/mL) and y is the absorbance at 700 nm. The Vc equivalent concentration (*C*, µg/mL) was calculated, and the reducing power was determined as follows:

(4) $$Reducing\;power\;(mg\;Vc\;equivalent/g\;DW)=\frac{C\times V\times D}{M\times 1000}$$

where *V* is the final volume of the extract (mL), *D* is the dilution factor, and *M* is the sample dry weight (g).

### 2.8. GC-IMS Analysis of Volatile Components

The analytical procedure employed a FlavourSpec^®^ GC-IMS system (G.A.S., Dortmund, Germany). 2 g of fresh fermented samples was heated at 60 °C for 20 min in a 20-mL headspace vial, and 500 μL of the vapor phase was auto-injected. The flow rate of the nitrogen carrier gas (≥99.999%) was programmed as: 2 mL/min (0–2 min), 10 mL/min (2–10 min), and 100 mL/min (10–40 min), following reported conditions [30]. Compound identification was based on dual matching of retention index (RI), relative drift time (Dt [RIPrel]), and NIST 2020 and VOCal databases. RI was derived via linear interpolation from a calibration curve constructed using six ketone homologs (C_4_–C_9_) run under the same conditions. The Reporter, Gallery Plot, and Dynamic principal component analysis (PCA) plug-ins enabled data visualization and comparative analysis.

### 2.9. Sensory Evaluation

All samples were stored at 4 °C overnight and then equilibrated to room temperature. The evaluation panel consisted of 20 healthy volunteers (10 males and 10 females, aged 20–35 years) recruited from Henan Institute of Science and Technology, all of whom provided written informed consent. Each panelist received protocol-specific training prior to the formal assessment. For each session, samples were sliced to uniform weight, placed on white plates, and assigned random three-digit codes. Panelists were asked to evaluate color, flavor, texture, and palatability using a five-point scale.

### 2.10. E-Tongue Analysis

Taste profiles of 48-h samples were analyzed using an SA402B electronic tongue system (Insent Co., Ltd., Atsugi, Japan). Samples were diluted 50-fold with distilled water before measurement. Each analysis consisted of 120 s sample detection, 40 s aftertaste acquisition, and 10 s sensor cleaning.

### 2.11. Untargeted Metabolomics Analysis

Following the method described by Vasilev et al. [31], each sample was weighed into a 2 mL tube, mixed with 600 µL MeOH (containing 20 ppm 2-amino-3-(2-chloro-phenyl)-propionic acid as internal standard (IS)), vortexed (30 s), homogenized with steel balls (55 Hz, 60 s), ultrasonicated (room temperature, 15 min), and centrifuged (12,000 rpm, 4 °C, 10 min). Supernatants were filtered (0.22 µm) for LC-MS analysis. A pooled QC sample was prepared from equal aliquots of all extracts to monitor analytical reproducibility.

LC-MS conditions: Separation used an ACQUITY UPLC HSS T3 column (150 × 2.1 mm, 1.8 µm) at 40 °C, with mobile phases of 0.1% formic acid in water (A) and acetonitrile (B), flow rate 0.25 mL/min, injection 5 µL. Gradient: 0–1 min, 2% B; 1–9.5 min, 2–50% B; 9.5–14 min, 50–98% B; 14–15 min, 98% B; 15–15.5 min, 98–2% B; 15.5–17 min, 2% B. Mass detection employed an LTQ Orbitrap XL with ESI± in Full MS-ddMS2 mode. Parameters: sheath/aux gas 45/15 arb, spray voltage ±4.80/−4.50 kV, capillary temperature 325 °C; MS1 resolving power 60,000 FWHM, ranges *m*/*z* 89–1000 (+) and 114–1000 (−); data-dependent MS/MS (4 scans/cycle) at 30% normalized collision energy (NCE) (normal resolution), dynamic exclusion 15 s. All data processing steps—including peak picking, alignment, and compound identification—were executed in Compound Discoverer 3.3, with metabolite assignments verified using the mzCloud, KEGG, and Panomics in-house metabolite standard databases.

### 2.12. Statistical Analysis

All experiments were carried out in triplicate, and the data are expressed as means with standard deviations (SD). All statistical analyses were performed with SPSS 17.0 (SPSS Inc., Chicago, IL, USA), using one-way ANOVA and Tukey’s HSD post-hoc test for multiple group comparisons, with the significance threshold set at *p* < 0.05. Multivariate analyses, including PCA and OPLS-DA, were run in SIMCA 14.1 (Umetrics, Umeå, Sweden), and the robustness of the models was verified through 200-fold permutation testing. Metabolites showing VIP scores > 1.0 and FDR-adjusted *p* < 0.05 were deemed to be significantly different. Heatmap visualization was performed with MetaboAnalyst 5.0, and pathway enrichment was explored using the KEGG database.

## 3. Results and Discussion

### 3.1. pH Value and Titratable Acidity During Fermentation

**Figure 1:**
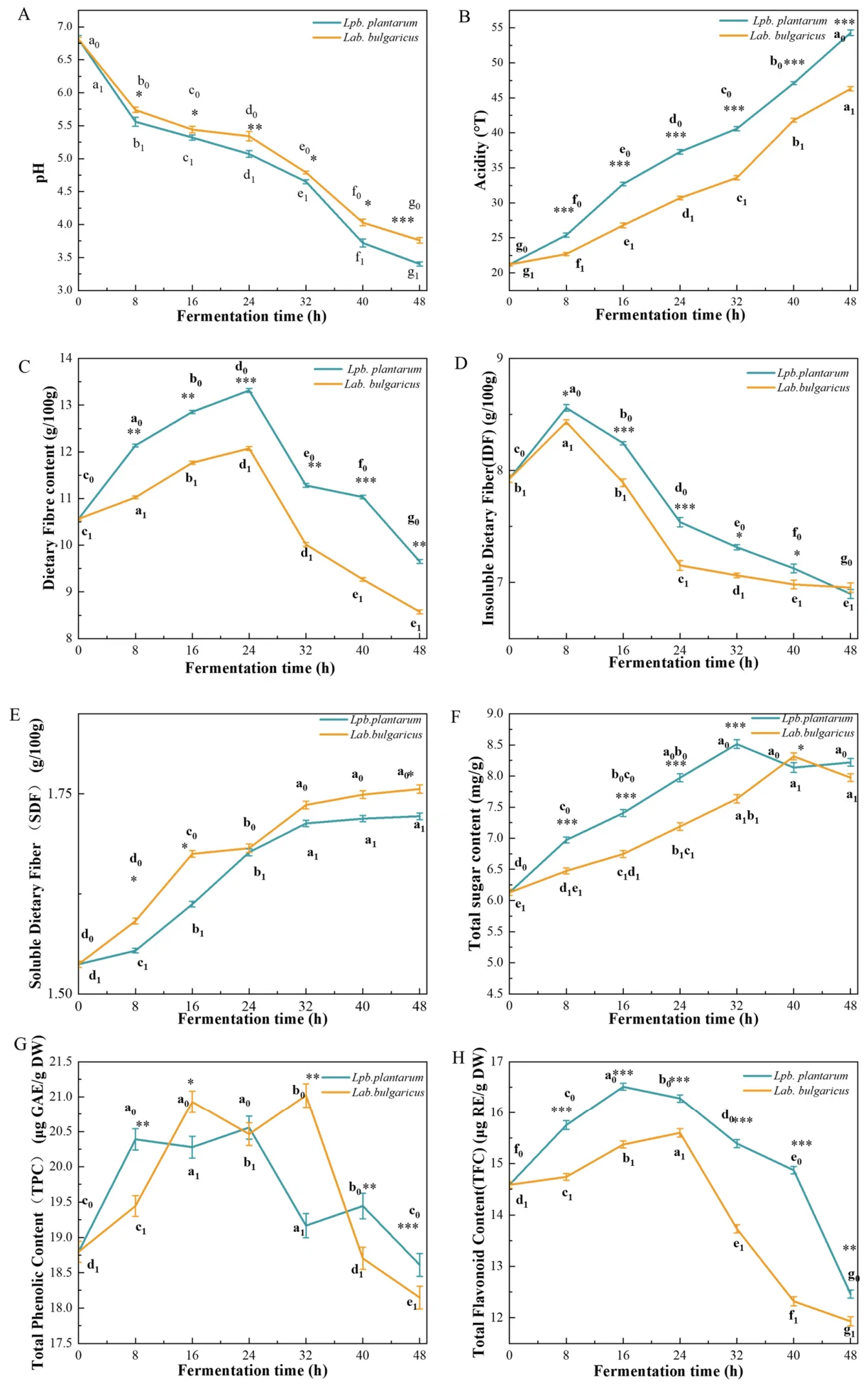
Effects of *Lactobacillus delbrueckii* subsp. *bulgaricus* (*Lab. bulgaricus*) and *Lactiplantibacillus plantarum* (*Lpb. plantarum*) fermentation on pH (**A**), acidity (**B**), total dietary fiber ((**C**), TDF), insoluble dietary fiber ((**D**), IDF), soluble dietary fiber ((**E**), SDF), total sugar content (**F**), total phenolic content ((**G**), TPC), and total flavonoid content ((**H**), TFC). For comparisons between *Lab. bulgaricus* and *Lpb. plantarum*, asterisks indicate significance levels (* *p* < 0.05, ** *p* < 0.01, *** *p* < 0.001), whereas different letters denote *p* < 0.05 for comparisons among other groups.

Fermentation with *Lab. bulgaricus* and *Lpb. plantarum* significantly affected the pH and titratable acidity of *F. velutipes* roots (Figure 1A,B). As fermentation proceeded, pH gradually decreased, whereas titratable acidity increased, with *Lpb. plantarum* showing a greater acidifying effect than *Lab. bulgaricus*. This difference may be attributed to the broader acid-producing capacity of *Lpb. plantarum*, which can convert diverse substrates into organic acids such as lactic, acetic, and phenyllactic acid [32]. In the early stage of fermentation, the rapid pH decline was accompanied by a marked accumulation of organic acids, reflecting vigorous LAB metabolic activity. As fermentation progressed, substrate depletion and the increasingly acidic environment likely slowed LAB growth and metabolism and eventually limited further acid production [33].

### 3.2. Changes in Dietary Fiber and Total Sugar Content During Fermentation

LAB fermentation significantly affected the dietary fiber composition of *F. velutipes* roots (Figure 1C). TDF content increased initially and then declined, reaching the maximum at 24 h in both *Lab. bulgaricus* and *Lpb. plantarum* fermentation groups. During fermentation, IDF (Figure 1D) generally decreased, whereas SDF (Figure 1E) progressively increased, suggesting a conversion of insoluble fiber into soluble fractions. This change may be related to fibrolytic enzymes, such as endoglucanases, which hydrolyze insoluble fibers into smaller soluble fragments, thereby potentially improving the physiological functions of dietary fiber [34].

A pattern of initial rise and subsequent fall was observed for total sugar content over the course of LAB fermentation (Figure 1F). In the *Lab. bulgaricus* group, the maximum sugar content appeared at 32 h, while the *Lpb. plantarum* group reached this peak at 40 h. Overall, *Lpb. plantarum* produced higher sugar levels than *Lab. bulgaricus* throughout fermentation. The prolonged fermentation period led to sustained consumption of soluble sugars and polysaccharide-derived carbohydrates by LAB, which is the probable reason for the subsequent drop in total sugar content [35,36].

### 3.3. Changes in Total Phenolic Content (TPC) and Total Flavonoid Content (TFC)

TPC and TFC initially increased and then declined with prolonged fermentation time (Figure 1G,H). The variation in TPC was more pronounced in the *Lab. bulgaricus* group than in *Lpb. plantarum*, whereas the opposite trend was observed for TFC. LAB fermentation is a complex process in which microorganisms release phenolic compounds from bound forms through enzymatic hydrolysis. At the onset of fermentation, the metabolic and enzymatic activities of LAB enhance biochemical reactions, thus favoring the accumulation of phenolic and flavonoid metabolites [37,38]. As fermentation progressed, reduced microbial activity and possible degradation or transformation of these compounds may have contributed to the decline in TPC and TFC. Similar changes have been reported in other LAB-fermented substrates [39,40].

### 3.4. Changes in Antioxidant Activity During Fermentation

**Figure 2:**
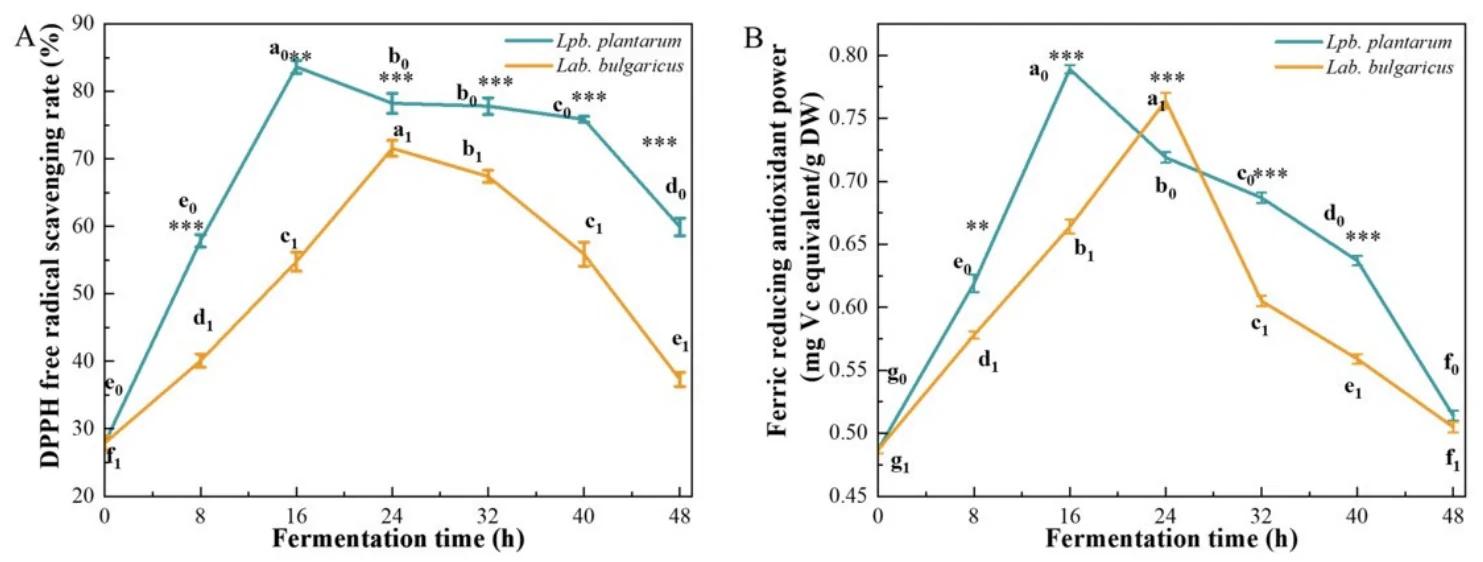
Effects of fermentation on DPPH radical scavenging activity (**A**) and ferric reducing ability (**B**) in *F. velutipes* roots during fermentation. Different letters indicate significant differences between groups (*p* < 0.05). Asterisks indicate significant differences between the *Lactobacillus delbrueckii* subsp. *bulgaricus* (*Lab. bulgaricus*) and *Lactiplantibacillus plantarum* (*Lpb. plantarum*) groups (* *p* < 0.05, ** *p* < 0.01, *** *p* < 0.001).

The antioxidant potential of the fermented substrates was primarily linked to their phenolic and flavonoid contents. Thus, the dynamic changes observed in these phytochemicals during LAB fermentation were considered a major factor affecting the DPPH scavenging and ferric-reducing activities [41]. The DPPH radical scavenging and ferric reducing assays were performed to assess the antioxidant capacity of *F. velutipes* roots throughout fermentation. Both antioxidant indices increased significantly at the early fermentation stage and then gradually declined with prolonged fermentation (Figure 2). The antioxidant capacity of the fermented materials was correlated with their phenolic and flavonoid contents, as evidenced by the consistency between the overall trends in antioxidant capacity and the TPC/TFC profiles [42].

However, antioxidant capacity remained relatively high at the later fermentation stage despite the reduction in TPC and TFC, indicating that additional antioxidant metabolites may also be involved [43]. LAB fermentation can facilitate the accumulation of low-molecular-weight metabolites with known antioxidant functions [44,45,46]. Our metabolomics data revealed significant accumulation of organic acids (e.g., citraconic acid), aromatic amino acid precursors (e.g., N-acetyl-L-phenylalanine), and reducing equivalents (e.g., nicotinic acid), which may collectively contribute to the enhanced antioxidant properties [47,48]. These results suggest that the antioxidant capacity of fermented samples is regulated by multiple metabolites rather than solely by TPC and TFC.

### 3.5. Analysis of Volatile Flavor Components in F. velutipes Roots

#### 3.5.1. Volatile Components in *F. velutipes* Roots Fermented by *Lab. bulgaricus* and *Lpb. plantarum*

**Figure 3:**
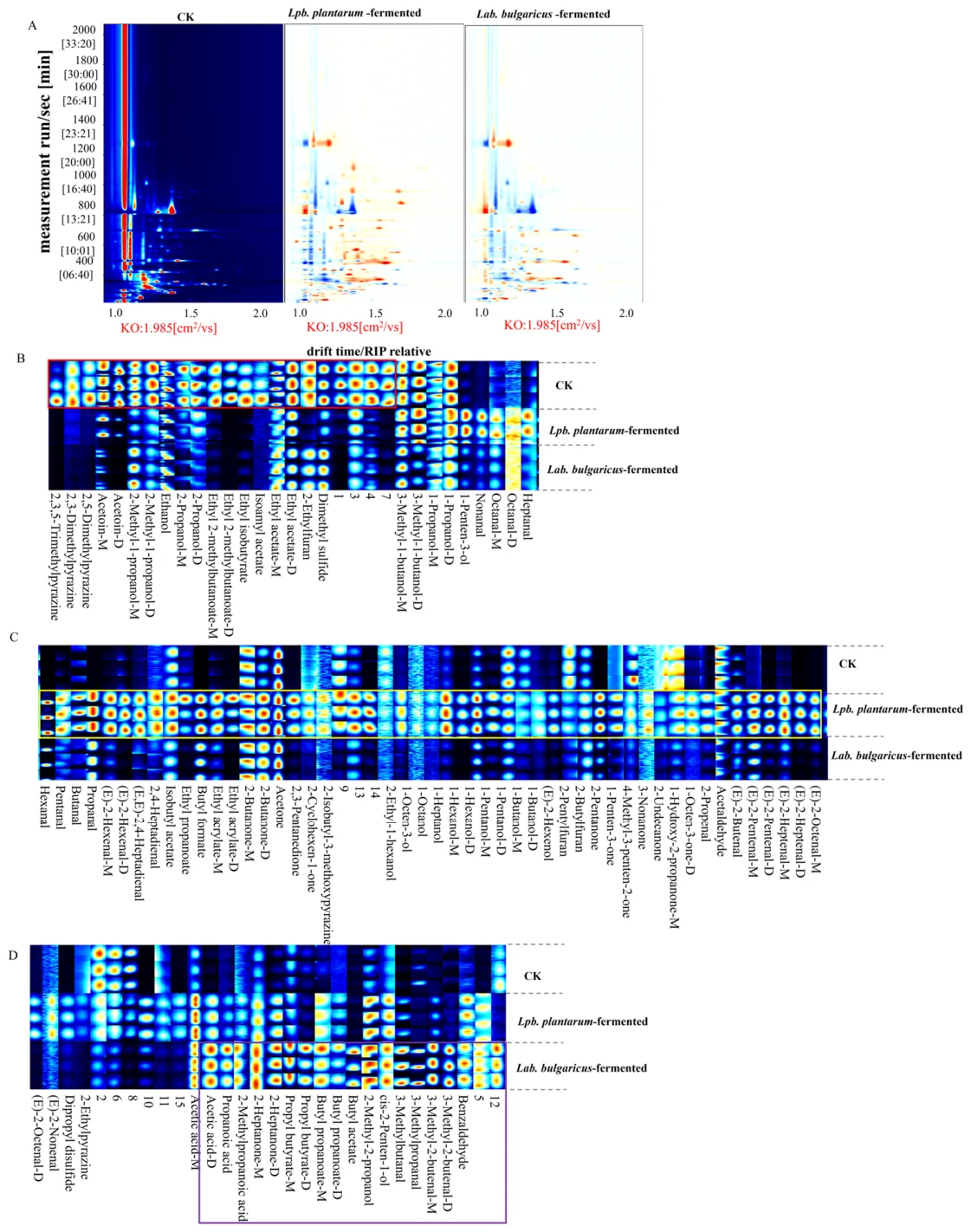
Compositional profiles of volatiles in the control (CK), *Lactiplantibacillus plantarum* (*Lpb. plantarum*)-fermented and *Lactobacillus delbrueckii* subsp. *bulgaricus* (*Lab. bulgaricus*)-fermented groups of *F. velutipes* roots. Plots of peak areas of selected signals (**A**) and fingerprints (**B**–**D**).

GC-IMS analysis of *F. velutipes* roots fermented with *Lpb. plantarum* or *Lab. bulgaricus*, alongside a non-fermented at 48 h as control (CK), was performed for volatile profiling. The two-dimensional topographic plots showed clear differences among the samples (Figure 3A). In the differential comparison plots, the CK served as the baseline, where red and blue coloration denoted elevated and diminished volatile signal intensities, respectively [49]. Notably, *Lpb. plantarum*-fermented samples exhibited marked changes within the retention time range of 400–800 s and drift time range of 1.2–1.6 s, suggesting that *Lpb. plantarum* fermentation substantially altered volatile profiles in *F. velutipes* roots.

The fingerprint patterns provided strong evidence for the pronounced volatile differences observed between the fermented samples and their unfermented counterparts (Figure 3B–D). A total of 112 volatile compounds were identified, including 23 alcohols, 17 ketones, 27 aldehydes, 16 esters, and 4 acids, among others (Table S5). Compared with the CK, fermentation markedly reduced the levels of several pyrazines (e.g., 2, 3, 5-trimethylpyrazine), esters (e.g., ethyl acetate), alcohols (e.g., ethanol and 2-propanol), furans (e.g., 2-ethylfuran) and sulfur-containing compounds (e.g., dimethyl sulfide) (Figure 3B, red area). *Lpb. plantarum* fermentation enhanced the production of aldehydes and alcohols, including nonanal, octanal, heptanal, hexanal, pentanal, butanal, propanal, *E*-2-hexenal, (*E*,*E*)-2,4-heptadienal, 3-methyl-1-butanol, and 1-penten-3-ol. *Lpb. plantarum* fermentation also led to a marked accumulation of organic acids, with acetic acid showing an approximately 12-fold increase compared to the control (CK) (Table S2) (Figure 3C, yellow area). *Lab. bulgaricus* fermentation similarly resulted in elevated organic acid levels, alongside higher abundances of certain ketones (e.g., 2-heptanone), esters (e.g., propyl butyrate, butyl acetate, and butyl propionate), alcohols (e.g., 2-methyl-2-propanol), and aldehydes (e.g., 3-methylbutanal, 2-methylpropanal, and benzaldehyde) (Figure 3D, purple area). Thus, both *Lpb. plantarum* and *Lab. bulgaricus* contributed substantially to organic acid production, although they differed quantitatively in their modulation of other volatile categories.

Certain volatiles—hexanal, (*E*)-2-hexenal, butyl formate, 2,3-pentanedione, and 1-hexanol—emerged solely in the fermented samples. These are characterized by green, fruity, creamy, floral, and fatty notes, likely contributing to the enhanced flavor complexity of *F. velutipes* roots following LAB fermentation. The two LAB strains also showed distinct but overlapping volatile-forming patterns. *Lpb. plantarum* fermentation promoted the formation of aldehydes and esters, whereas *Lab. bulgaricus* fermentation showed relatively higher abundances of ketones and some ester species. Notably, organic acid accumulation, particularly acetic acid, was not exclusive to either strain; both strains exhibited substantial increases, with the *Lab. bulgaricus* group showing the highest levels among all samples, approximately 1.5-fold higher than the *Lpb. plantarum* group (Table S2). The observed differences between the two strains may be partly attributed to their distinct metabolic capacities, including enzymatic activities involved in fatty acid oxidation, amino acid conversion, glycolytic pathways, and esterification [50].

Multivariate analysis was performed to identify the key volatile compounds contributing to sample discrimination. Features with VIP > 1.0 were regarded as important contributors to the observed sample clustering [51]. Five compounds, namely 2,3-pentanedione, butyl formate, ethyl propenoate, heptenal, and 1-hexanol-D were identified as key differential volatiles (Table S2). Their peak intensities increased after fermentation, particularly in the *Lpb. plantarum* fermentation group, highlighting the marked impact of LAB fermentation on the volatile profile of *F. velutipes* roots.

Overall, pyrazines were the dominant volatile compounds in the CK group, especially 2,3,5-trimethylpyrazine, 2,3-dimethylpyrazine, and 2,5-dimethylpyrazine, followed by alcohols, whereas esters and acids were present at relatively low levels. After fermentation, organic acids such as acetic and propionic acids accumulated substantially in both fermentation groups, reflecting the likely involvement of LAB-mediated metabolism. These acids can be converted into esters, ketones, alcohols, and aldehydes. Overall, LAB fermentation, particularly with *Lpb. plantarum*, substantially modified the volatile composition of *F. velutipes* roots, with each strain exerting a distinct influence on the volatile profile.

#### 3.5.2. Multivariate Statistical Analysis of Volatile Components

**Figure 4:**
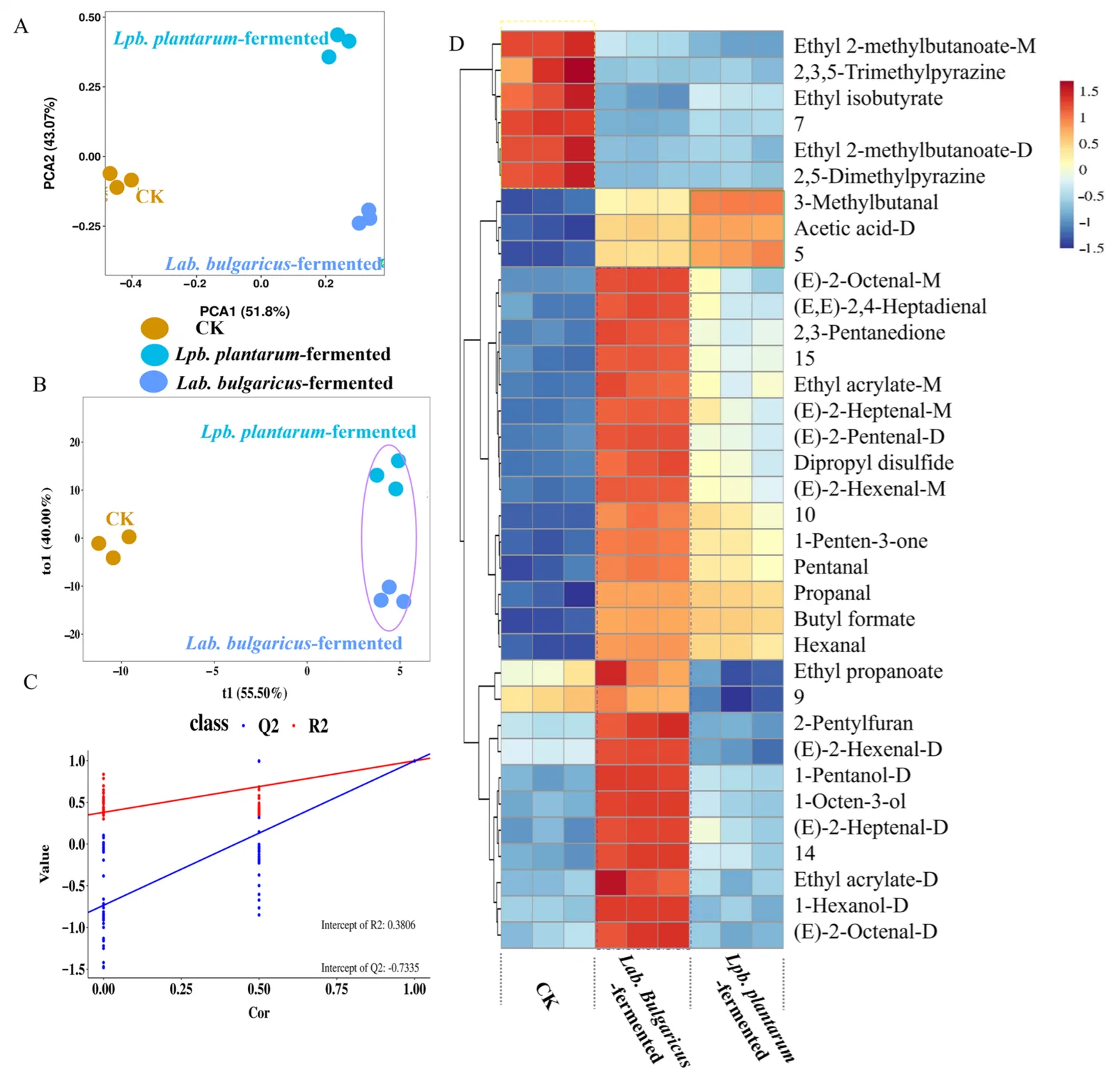
PCA score (**A**), OPLS-DA (**B**,**C**), and heatmap (**D**) of volatile components. The yellow area represents substances with higher content in the control (CK) group, the green area represents substances with higher content in *Lpb. plantarum*-fermented group, and the purple area represents substances with higher content in group *Lab. bulgaricus*-fermented.

To visualize the volatile profile differences among the sample groups, PCA was applied. The first two PCs together explained 94.87% of the total variance, with PC1 and PC2 contributing 51.8% and 43.07%, respectively. The PCA score plot showed clear separation among the control (CK), *Lpb. plantarum*-fermented, and *Lab. bulgaricus*-fermented groups (Figure 4A), indicating that LAB fermentation substantially altered the volatile makeup of *F. velutipes* roots. The distinct distribution of the *Lpb. plantarum* and *Lab. bulgaricus* groups further suggested that the volatile profile was influenced by the choice of starter culture.

An OPLS-DA model was then established to further evaluate differences among the three groups. Clear separation was observed in the OPLS-DA score plot (Figure 4B), indicating significant differences in volatile profiles. Model validation was performed via 200 permutation tests, which gave R^2^ = 0.998, Q^2^ = 0.996, and a Q^2^ intercept of −0.7335 (Figure 4C), indicating good stability and no obvious overfitting. The heatmap of volatile compounds with VIP values > 1 showed distinct differences in signal intensity among groups (Figure 4D; Table S2). Notably, the *Lpb. plantarum*-fermented group exhibited higher intensities of several key volatiles than the CK and *Lab. bulgaricus*-fermented groups, suggesting that *Lpb. plantarum* fermentation may have a more pronounced effect on the accumulation of these specific compounds.

Fatty acid oxidation, amino acid degradation, and glycoside hydrolysis may all contribute to the production of key volatile compounds during fermentation. Linoleic acid oxidation can produce hexanal, 1-octen-3-ol, (*E*)-2-heptenal, (*E*)-2-octenal, and (*E*)-2-nonenal, whereas linolenic acid degradation can generate (*Z*)-3-hexen-1-ol and (*E*)-2-hexenal, which are commonly related to grassy and fruity aromas [48]. Thus, enzymatic oxidation of lipids may be partly responsible for the increased hexanal content observed after fermentation. In addition, 2,3-pentanedione, associated with buttery and toffee-like notes associated with fruity aroma, may be formed through enzymatic reactions linked to glycoside hydrolysis and amino acid metabolism [52].

To further clarify the metabolic basis of these volatile changes, LC-MS analysis was conducted to identify related non-volatile metabolites.

### 3.6. Untargeted Metabolomics Analysis

Building on the above observations, LAB fermentation also markedly affected the non-volatile metabolite profiles of *F. velutipes* roots. To gain deeper insight into the non-volatile metabolite changes following fermentation, we conducted untargeted metabolomic profiling via LC-MS. A total of 48 metabolites were annotated across the control (CK) and fermented samples. The identified metabolites fell into 12 broad classes, namely amino acids, organic acids and derivatives, carbohydrates and derivatives, lipids and lipid-like molecules, alcohols and polyols, amines, terpenoids, phenolic compounds, aldehydes, alkane derivatives, ketones, and other compounds.

**Figure 5:**
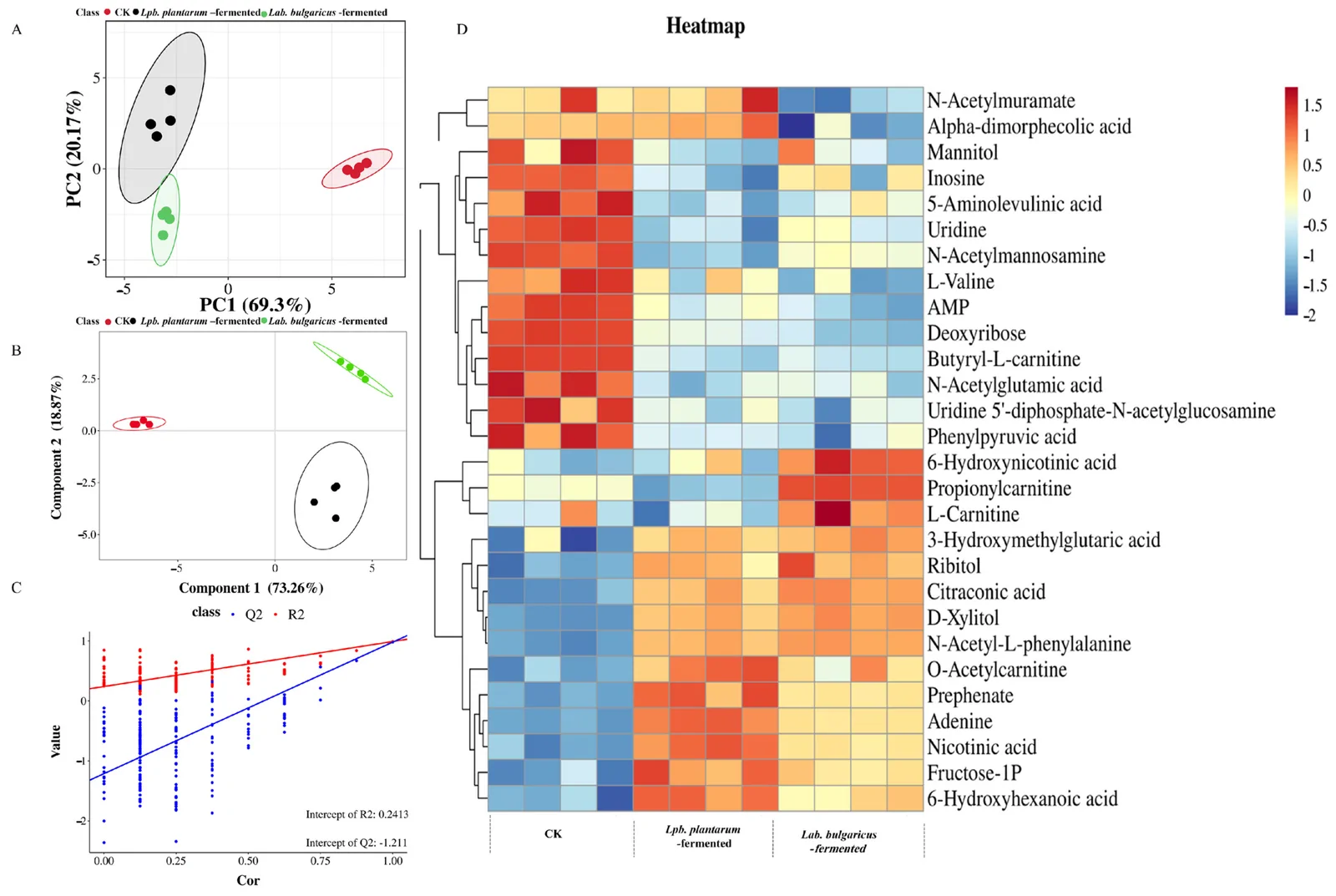
Comparison of metabolites. PCA score chart (**A**). OPLS-DA score chart (**B**). OPLS-DA (**C**) replacement test diagram. Differential metabolite clustering heat map (**D**).

To visualize the metabolic signatures distinguishing the various treatments, PCA was applied. The first two principal components jointly captured 89.47% of the total variance, with PC1 and PC2 accounting for 69.3% and 20.17%, respectively (Figure 5A). The PCA score plot clearly separated the control (CK) samples from the fermented groups and also distinguished the *Lpb. plantarum*- and *Lab. bulgaricus*-fermented samples, suggesting that the metabolite profiles were influenced by the choice of starter culture. The OPLS-DA model also supported this group discrimination (Figure 5B), with good reliability and predictive ability (R^2^Y = 0.991, Q^2^ = 0.981), and a negative Q^2^ intercept, suggesting no obvious over-fitting. Metabolites with VIP > 1.0 and FDR-adjusted *p* < 0.05 were defined as differential (Table S3).

The cluster heatmap further confirmed distinct metabolic patterns among groups (Figure 5D). Compared with the CK, *Lab. bulgaricus* fermentation significantly decreased the levels of N-acetylmuramate, linoleic acid, and mannitol (FDR-adjusted *p* < 0.05), while increasing those of D-xylitol, N-acetyl-L-phenylalanine, and 3-hydroxymethylglutaric acid (FDR-adjusted *p* < 0.05). A significant reduction in linoleic acid, L-valine, and phenylpyruvic acid was observed in the *Lpb. plantarum*-fermented group (FDR-adjusted *p* < 0.05), whereas nicotinic acid, prephenate, and N-acetyl-L-phenylalanine showed significant accumulation (FDR-adjusted *p* < 0.05). The enhanced antioxidant activity of the fermented samples may be partly associated with the accumulation of N-acetyl-L-phenylalanine, D-xylitol, nicotinic acid, adenine, and citraconic acid, among other metabolites. These results suggest that organic acids, amino acids, carbohydrates, and nucleoside-related compounds were among the metabolite groups substantially affected by fermentation with *Lpb. plantarum* and *Lb. bulgaricus*.

Volcano plot analysis further revealed substantial metabolic shifts after fermentation (Figure S3). In comparison with the control (CK), the number of upregulated and downregulated metabolites was 19 and 56, respectively, for the *Lpb. plantarum*-treated group, and 23 and 23 for the *Lab. bulgaricus*-treated group. The comparison between *Lpb. plantarum*- and *Lab. bulgaricus*-fermented samples identified 12 upregulated and 42 downregulated metabolites, further suggesting that the two LAB strains exerted distinct metabolic effects.

**Figure 6:**
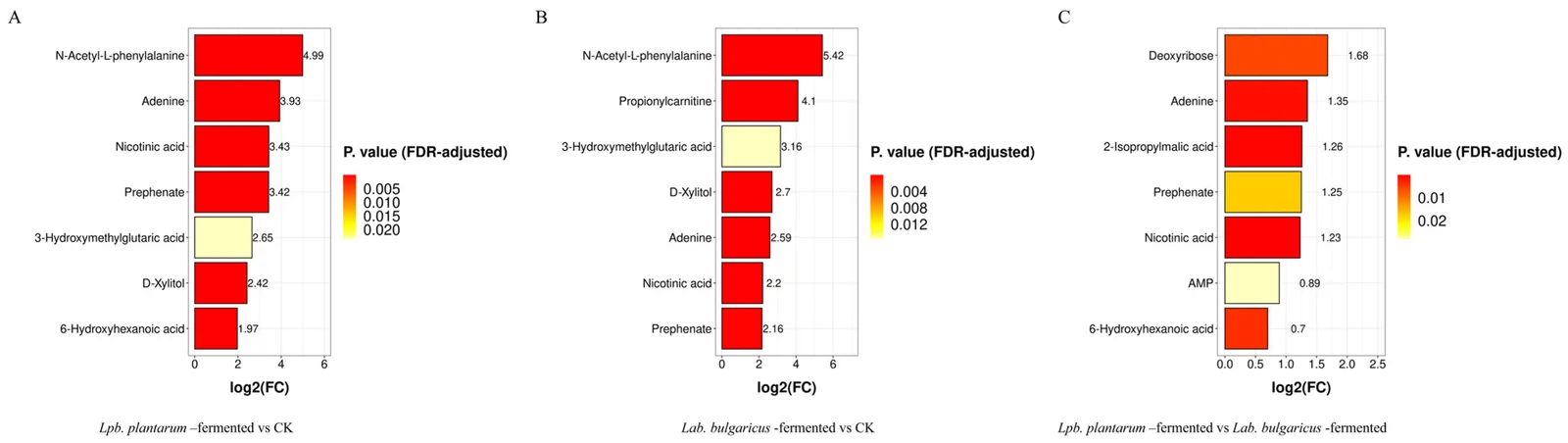
Significant differential metabolites identified in the group comparisons. *Lpb. plantarum* vs. control (CK) (**A**), *Lab. bulgaricus* vs. control (CK) (**B**), and *Lpb. plantarum* vs. *Lab. bulgaricus* group (**C**).

To identify the most relevant differential metabolites, fold-change thresholds were combined with VIP values. For the comparisons of *Lpb. plantarum* or *Lab. bulgaricus* against the control (CK), FC > 3 and VIP > 1 were used, whereas FC > 1.5 and VIP > 1 were applied for the *Lpb. plantarum* versus *Lab. bulgaricus* comparison. In the *Lpb. plantarum*-fermented group, the major differential metabolites included carboxylic acids and derivatives, alcohols, and aromatic amino acid-related compounds. Representative metabolites with significant increases included N-acetyl-L-phenylalanine, adenine, nicotinic acid, prephenate, D-xylitol, and 6-hydroxyhexanoic acid (Figure 6A). Among these, nicotinic acid and D-xylitol are associated with antioxidant activities potentially through enhancing reducing power and free radical scavenging, among other mechanisms [53,54].

The metabolite profile of the *Lab. bulgaricus*-fermented group was dominated by amino acids, carboxylic acids, alcohols, and carnitine derivatives. Among these, the most abundant compounds were N-acetyl-L-phenylalanine, propionylcarnitine, D-xylitol, adenine, nicotinic acid, and prephenate (Figure 6B). Propionylcarnitine is of particular interest because of its reported involvement in mitochondrial fatty acid oxidation and antioxidant defense under oxidative stress [55]. The comparison between *Lpb. plantarum*-fermented and *Lab. bulgaricus*-fermented groups further identified deoxyribose, 6-hydroxyhexanoic acid, and citraconic acid as major differential metabolites (Figure 6C), implying that these compounds may contribute to the metabolic divergence between the two LAB strains.

Overall, LC-MS-based metabolomics revealed that fermentation by *Lpb. plantarum* and *Lab. bulgaricus* substantially altered the non-volatile metabolite profile of *F. velutipes* roots. The accumulation of amino acids, organic acids, carbohydrates, and nucleosides may be associated with both the improved biological activity and the production of volatile odor-active compounds in the fermented samples. These metabolites can serve as precursors or intermediates in fatty acid oxidation, amino acid degradation, Strecker degradation, Maillard reactions, and microbial biotransformation, thereby contributing to the generation of various GC-IMS-detected aroma-active substances, including aldehydes, alcohols, ketones, and esters.

### 3.7. Metabolic Pathway Analysis

**Figure 7:**
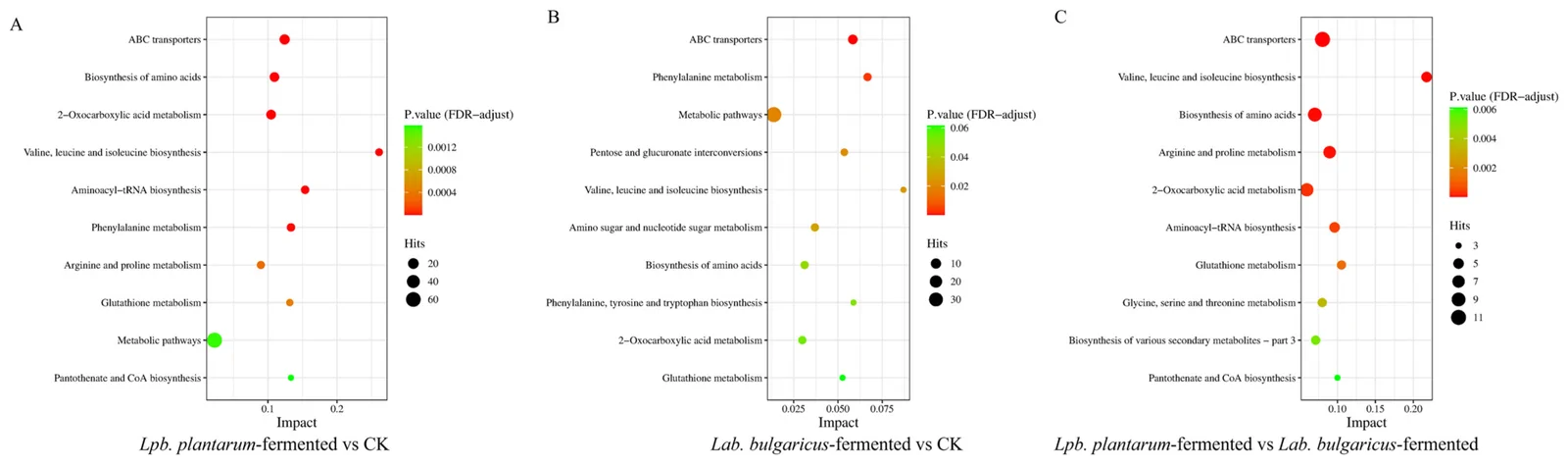
KEGG enrichment pathways of differential metabolites in group comparisons. *Lpb. plantarum* vs. control (CK) group (**A**), *Lab. bulgaricus* vs. control (CK) group (**B**), *Lpb. plantarum* vs. *Lab. bulgaricus* group (**C**).

To gain further insight into the metabolic mechanisms associated with by LAB fermentation in *F. velutipes* roots, KEGG pathway enrichment analysis was conducted. Significant pathway alterations were observed in all comparison groups. In the *Lpb. plantarum*-fermented vs. CK comparison, the top 10 pathways are shown in Figure 7A (FDR-adjusted *p* < 0.05). The major enriched pathways included 2-Oxocarboxylic acid metabolism, Valine, leucine, and isoleucine biosynthesis, and aminoacyl-tRNA biosynthesis. This suggests that the metabolic perturbation associated with *Lpb. plantarum* fermentation is substantial relative to the control (CK) group.

In the *Lab. bulgaricus*-fermented vs. CK comparison, 10 significantly enriched pathways were shown in Figure 7B (FDR-adjusted *p* < 0.05), mainly involving phenylalanine metabolism and ABC transporters.

Direct comparison between the *Lpb. plantarum*-fermented and *Lab. bulgaricus*-fermented groups identified the top 10 pathways, as shown in Figure 7C (FDR-adjusted *p* < 0.05). The ABC transporters were among the significantly enriched pathways. In addition, the pathways involved in the biosynthesis of amino acids and 2-Oxocarboxylic acid metabolism were also enriched.

Overall, pathway enrichment analysis suggested that *Lpb. plantarum* fermentation associated with robust Valine, leucine, and isoleucine biosynthesis and 2-oxocarboxylic acid metabolism. In contrast, *Lab. bulgaricus* showed more prominent perturbations in phenylalanine metabolism, with less pronounced involvement in valine, leucine, and isoleucine biosynthesis. The observed inter-strain differences were partly associated with variations in ABC transporter functionality and valine, leucine, and isoleucine biosynthetic capacity.

### 3.8. Sensory Evaluation and Electronic Tongue Analysis

**Figure 8:**
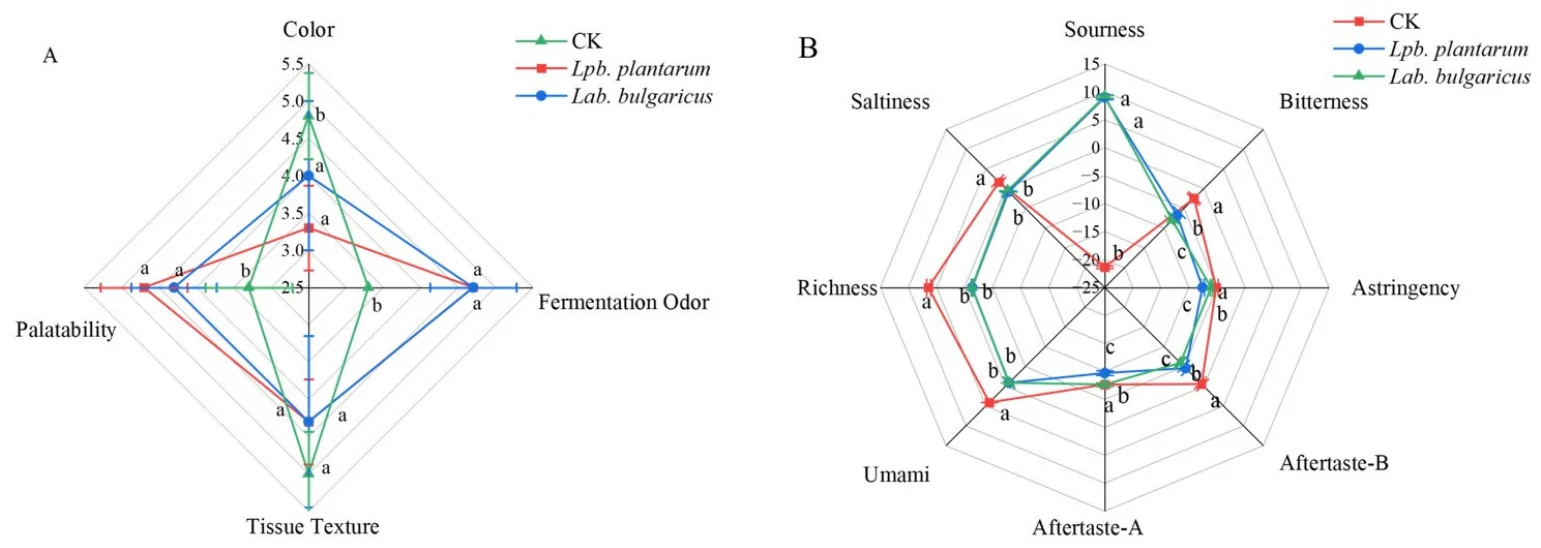
Radar charts of sensory evaluation (**A**) and electronic tongue measurements (**B**). The different letters denote *p* < 0.05 for comparisons among other groups.

Sensory evaluation and E-tongue analysis were conducted to assess the effects of LAB fermentation on the sensory characteristics of *F. velutipes* roots. The sensory results showed that fermentation improved odor and palatability, although color and tissue texture scores decreased slightly (Figure 8A).

E-tongue analysis further revealed the changes in taste profiles after fermentation (Figure 8B). Compared with the control (CK), both *Lpb. plantarum*-fermented and *Lab. bulgaricus*-fermented samples showed higher sourness, which may be partly attributable to the accumulation of organic acids during fermentation. In contrast, bitterness, astringency, and bitter aftertaste were generally reduced, suggesting that fermentation contributed to the attenuation of undesirable taste attributes. The control (CK) group showed relatively higher umami and richness, suggesting that certain native taste-active compounds were likely consumed or transformed during fermentation. Differences between *Lpb. plantarum* and *Lab. bulgaricus* were also observed, suggesting that the two starter cultures exerted distinct effects on taste-related metabolites.

Overall, the sensory and E-tongue results indicated that *Lpb. plantarum* and *Lab. bulgaricus* fermentation enhanced the palatability of *F. velutipes* roots, likely through a combination of enhanced aroma, increased sourness, and reduced bitterness and astringency. These changes were consistent with the metabolomic results, especially the alterations in organic acids, amino acids, sugar alcohols, and phenolic compounds.

## 4. Conclusions

This study showed that fermentation with *Lab. bulgaricus* and *Lpb. plantarum* improved the odor and palatability while reducing the bitterness of *F. velutipes* roots. The partial conversion of insoluble fiber to soluble fiber was observed during fermentation with *Lpb. plantarum* and *Lab. bulgaricus*, which also altered the levels of sugars, organic acids, phenolic acids, and other bioactive compounds. These compositional changes were correlated with enhanced antioxidant activity, improved flavor complexity, and better palatability.

Starter-culture-dependent metabolic regulation was associated with the observed improvements in odor and bitterness of fermented samples. *Lpb. plantarum* fermentation was associated with metabolic alterations including sugar alcohols, hydroxy fatty acids, esters, and amino acid derivatives, which correlated with improved antioxidant defenses and enhanced flavor complexity, whereas *Lab. bulgaricus* fermentation showed a more pronounced enrichment of organic acids. Combined GC-IMS and LC-MS analyses suggested that amino acid metabolism, fatty acid degradation, and organic acid metabolism pathways were potentially involved in the formation of volatile flavor compounds. However, as no formal integrative analysis was performed, these observed associations should be considered exploratory and warrant further investigation. Sensory evaluation and E-tongue analysis further indicated that *Lpb. plantarum* and *Lab. bulgaricus* fermentations improved fermentation-derived aroma and palatability, while increasing sourness and reducing bitterness and astringency. It should be noted that the present study employed only one strain each of *Lactobacillus delbrueckii* subsp. *bulgaricus* and *Lactiplantibacillus plantarum*. The observed differences, therefore, represent only the phenotypic variations between these two specific starter cultures under the experimental conditions employed. Given the potential metabolic diversity among strains within a species, the present results should not be directly extrapolated to generalized conclusions at the species level. Future studies aiming to establish species-level patterns would require the inclusion of multiple representative strains per species for systematic comparison and cross-validation. Collectively, these results suggest that *Lpb. plantarum* and *Lab. bulgaricus* fermentations are a promising route for upcycling *F. velutipes* root residues, and may pave the way for the design of fermented mushroom preparations with customizable functional and organoleptic properties.

## Data Availability

The original contributions presented in the study are included in the article/Supplementary Materials, further inquiries can be directed to the corresponding author.

## Supplementary Materials

The following supporting information can be downloaded at: https://www.mdpi.com/article/10.3390/foods15172973/s1, Figure S1: Glucose standard curve, Figure S2: Vc standard curve, Figure S3: Volcano plots for *Lpb. plantarum* vs. control (CK) (A), *Lab. bulgaricus* vs. control (CK) (B), and *Lpb. plantarum* vs. *Lab. bulgaricus* groups (C), Table S1: Sensory evaluation standards for *Flammulina velutipes* roots, Table S2: Volatile substances in control (CK), *Lpb. plantarum*-fermented and *Lab. bulgaricus*-fermented samples (VIP > 1, *p* (FDR-adjusted) < 0.05), Table S3: Category A (genuine endogenous) differential metabolites in *Lpb. plantarum* and *Lab. bulgaricus*-fermented samples compared to the control (CK), Table S4: Category B (exogenous-derived residues) detected in the samples, Table S5: Volatile substances in control (CK), *Lpb. plantarum*-fermented and *Lab. bulgaricus*-fermented sample.
