# Supplementary material for: Starter-Culture-Dependent Effects of Lactiplantibacillus plantarum and Lactobacillus delbrueckii subsp. bulgaricus Fermentation on Nutritional Quality, Flavor Characteristics and Metabolite Profiles of Flammulina velutipes Roots
Source: Foods. 2026 Aug 24;15(17):2973. doi: 10.3390/foods15172973 (PMC13565036; doi:10.3390/foods15172973)
Supplement: Supplementary file 1 [file foods-15-02973-s001.zip › foods-4441209-supplementary.pdf]

Supplementary Materials

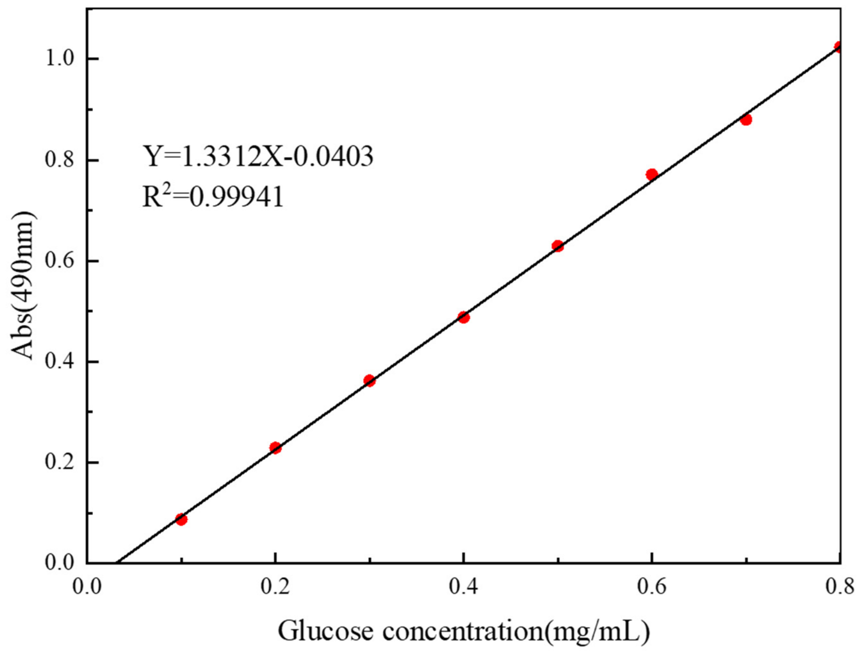

Figure S1. Glucose standard curve.

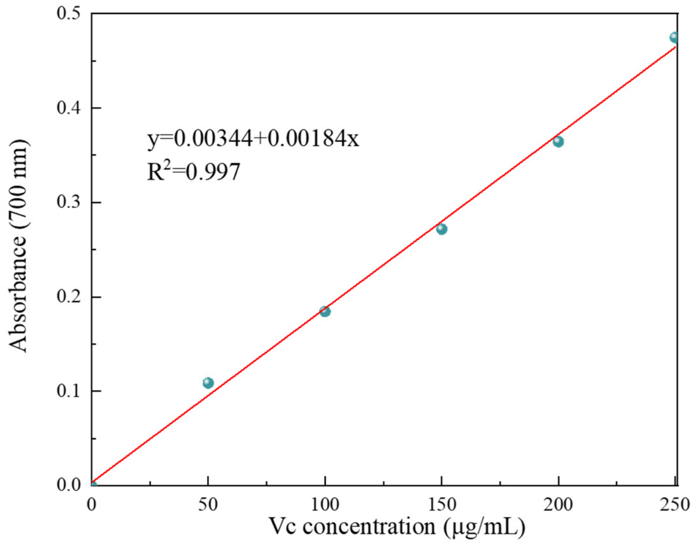

Figure S2. Vc standard curve.

Table S1. Sensory evaluation standards for Flammulina velutipes roots.

Items	Evaluation Criteria	points
color	Uniform light brown, no fermentative browning or dark spots	5
	Basically uniform brown, with slight local fading or discoloration	3～4
	Obvious dark brown/fermentative browning, sticky or moldy appearance	1～2
Fermentation Odor	Strong and pleasant fermented aroma (e.g., nutty, ester-like), no earthy or off-odor	5
	Moderate fermented aroma, with weak earthy or slightly off-odor	3～4
	Weak or no fermented aroma, obvious earthy/rancid or putrid odor	1～2
Tissue Texture	Compact and tough, no softening or fiber loosening	5
	Slightly soft, with local fermentative softening but still intact	3～4
	Obviously soft, loose fibers, sticky or completely mushy	1～2
Palatability	No residue when chewed, coordinated and pleasant fermented flavor	5
	Slight residue, acceptable flavor with mild bitter/astringent notes	3～4
	Obvious residue, strong bitter and astringent taste, unpleasant aftertaste	1～2

Table S2. Volatile substances in control (CK), *Lpb.plantarum*-fermented and *Lab.bulgaricus*-fermented samples (VIP > 1, $p < 0.05$).

Name	CAS#	RI	Rt(s)	Dt [RIPrel]	Peak Volume			VIP
					CK	***Lpb.plantarum***	***Lab.bulgaricus***	
2,3-Pentanedione	C600145	1051.8	436.781	1.31688	39.696±3.275	1054.575±84.83	152.093±23.598	2.791
Butyl formate	C592847	994.6	371.707	1.51209	79.905±11.724	7960.759±239.399	4649.697±399.343	2.628
Ethyl acrylate-D	C140885	1053.6	439.018	1.40621	65.228±8.562	1262.554±459.573	72.133±16.445	2.328
(E)-2-Heptenal-D	C18829555	1329.1	878.496	1.67014	95.736±26.304	4142.704±202.638	263.945±115.273	2.234
1-Hexanol-D	C111273	1368.8	956.435	1.64786	165.842±14.501	2776.077±31.94	140.993±20.411	1.999
1-Penten-3-one	C1629589	1024.6	404.489	1.30678	231.944±22.114	6353.724±112.844	2096.072±297.263	1.956
Hexanal	C66251	1084.7	479.274	1.55688	350.908±46.088	13894.36±206.932	6761.658±784.664	1.936
(E)-2-Pentenal-D	C1576870	1136	568.731	1.36088	326.711±35.201	6868.264±129.495	1147.06±161.517	1.848
Ethyl 2-methylbutanoate-D	C7452791	1050.1	434.68	1.64429	816.39±219.225	31.594±2.217	35.949±5.759	1.776
Propanal	C123386	785.1	234.235	1.1506	340.648±93.89	7216.542±62.168	4740.971±278.187	1.760
(E)-2-Octenal-M	C2548870	1437.4	1108.099	1.33554	231.979±5.871	3726.127±78.388	617.277±244.796	1.663
Dipropyl disulfide	C629196	1416	1058.43	1.2672	83.516±2.7	712.084±70.227	224.135±34.681	1.543
Ethyl propanoate	C105373	958.2	336.282	1.44785	274.986±68.617	709.124±271.835	74.471±15.166	1.517
1-Pentanol-D	C71410	1258.7	763.55	1.51459	214.022±18.006	1545.036±33.963	299.979±16.92	1.470
Acetic acid-D	C64197	1502.4	1273.86	1.16016	592.904±87.883	7023.553±426.527	10332.205±178.116	1.429
Ethyl isobutyrate	C97621	963.9	341.569	1.55491	2500.626±694.857	171.703±20.189	332.703±19.96	1.364
(E)-2-Heptenal-M	C18829555	1328.6	877.534	1.25606	362.028±19.059	4255.461±44.918	1255.602±342.968	1.355
Ethyl acrylate-M	C140885	1053.9	439.385	1.13297	208.82±6.172	1861.786±139.086	596.814±91.46	1.337
Pentanal	C110623	984.3	361.132	1.43022	115.652±19.742	1413.783±35.734	599.038±55.825	1.334
2,3,5-Trimethylpyrazine	C14667551	1393.7	1008.96	1.15406	1970.764±1104.255	111.452±5.512	113.241±10.575	1.330
Ethyl 2-methylbutanoate-M	C7452791	1050.8	435.6	1.25145	1674.35±151.458	326.392±27.329	221.305±13.413	1.305
(E)-2-Hexenal-M	C6728263	1222.1	713.958	1.17993	156.817±7.153	1775.949±23.911	512.297±64.311	1.304
2,5-Dimethylpyrazine	C123320	1351.3	921.37	1.11131	496.239±92.192	57.011±3.692	62.445±2.832	1.137
2-Pentylfuran	C3777693	1231.9	726.94	1.25145	612.623±21.729	2592.262±294.302	428.682±26.694	1.110
(E)-2-Hexenal-D	C6728263	1222.1	713.958	1.50859	451.135±15.113	3094.844±82.989	176.351±27.923	1.075

3-Methylbutanal	C590863	920.7	303.5	1.39495	190.054±24.129	1091.342±57.115	2536.618±59.902	1.074
(E)-2-Octenal-D	C2548870	1437.4	1108.099	1.81803	154.512±21.03	671.921±51.201	134.142±11.976	1.012
(E,E)-2,4-Heptadienal	C4313035	1516.7	1313.364	1.20321	180.273±24.769	1295.051±46.259	385.812±76.692	1.009
1-Octen-3-ol	C3391864	1485.8	1229.318	1.1661	129.133±8.053	623.162±11.126	157.144±14.897	1.003

Table S3. Category A (genuine endogenous) differential metabolites in *Lpb. plantarum*- and *Lab. bulgaricus*-fermented samples compared to the control (CK).

name	Rt(s)	formula	*Lpb.plantarum* **(means± SD)**	*Lab.bulgaricus* **(means± SD)**	CK **(means± SD)**	***P***.value (FDR-adjust)	VIP	Category
AMP	149.2	$C_{10}H14N5O_7P$	80671.73±18909.45b	43616.44±16604.78c	381803.4±59217.70a	$3.66E^{-06}$	1.92	A
Deoxyribose	92.5	$C_5H_{10}O_4$	1810973±414712.70b	564135.8±379719.80c	71645377±8740735.99 a	$3.51E^{-08}$	1.90	A
Citraconic acid	157.6	$C_5H_6O_4$	5256432±353757.89b	5819290±169341.30a	2745050±219329.51c	$5.72E^{-08}$	1.82	A
D-Xylitol	103.1	$C_5H_{12}O_5$	$1.02e^{+08}$±12246996.48b	$1.24e^{+08}$±11158306a	19138165±901407.55c	$4.62E^{-10}$	1.81	A
N-Acetyl-L-phenylalanine	457.5	$C_{11}H_{13}NO_3$	745962±172914.00b	1001600±113180.80a	23460.39±4947.79c	$7.35E^{-10}$	1.79	A
Uridine 5'-diphosphate-N-acetylglucosamine	180.4	$C_{17}H_{27}N_3O_{17}P_2$	1259269±164082.69b	1141399±213784c	2412154±428639.74a	0.000458	1.73	A
Ribitol	92.6	$C_5H_{12}O_5$	$3e^{+08}$±28429297.57b	$3.21e^{+08}$±33043407a	$1.8e^{+08}$±12041303.9c	$1.43E^{-05}$	1.72	A
L-Valine	134.6	$C_5H_{11}NO_2$	47584863±11900974.13b	33072093±8777333c	80086885±13718883.3 a	0.001256	1.72	A
Phenylpyruvic acid	443.6	$C_9H_8O_3$	50462.14±1347.34b	45993.53±14198.20c	132102.7±29736.32a	0.000299	1.71	A
Butyryl-L-carnitine	296	$C_{11}H_{21}NO_4$	60643.82±36525.77b	60514.25±26825.97c	4571270±69483.31a	$4.32E^{-05}$	1.70	A
6-Hydroxynicotinic acid	177.1	$C_6H_5NO_3$	498474.3±111957.32b	794077.1±7670.85a	436981.4±68737.18c	0.00132	1.69	A
3-Hydroxymethylglutaric acid	307.3	$C_6H_{10}O_5$	864734.6±209092.83b	1235665±315588.80a	138216.1±18503.87c	0.000705	1.66	A
N-Acetylglutamic acid	207.5	$C_7H_{11}NO_5$	88436.75±26159.59c	105287.9±25732.37b	459627±128419.77a	$3.8E^{-05}$	1.55	A
N-Acetylmuramate	149.2	$C_{11}H_{19}NO_8$	282031.9±49778.63a	167002.5±19956.79c	269941±49966.08b	0.001746	1.46	A
Alpha-dimorphecolic acid	847	$C_{18}H_{32}O_3$	584855±163700.92a	99998.06±79398.46c	422633.2±39051.31b	0.000339	1.46	A
Uridine	220.5	$C_9H_{12}N_2O_6$	9044.2±5419.28c	22296.84±10129.45b	308443.4±5661.05a	0.000236	1.36	A
Fructose-1P	148.4	$C_6H_{12}O_6P$	12484958±1941411.04a	9308908±723302.2b	4853199±1093069.94c	$2.97E^{-05}$	1.31	A
Adenine	143.9	$C_5H_5N_5$	13063001±2185437.99a	5132238±215040.8b	855325.2±104948.40c	$5.75E^{-06}$	1.29	A
5-Aminolevulinic acid	149.2	$C_5H_9NO_3$	62752.98±10385.62c	84556.05±15693.6b	179649.4±32570.43a	$4.87E^{-05}$	1.29	A
Nicotinic acid	193	$C_6H_5NO_2$	3444906±601146.15a	1468299±121575.2b	319019.2±89484.89c	$1.07E^{-07}$	1.27	A
O-Acetylcarnitine	150.5	$C_9H_{18}NO_4$	20563137±3263369.90a	15376215±3596497b	7727243±1029268.73c	0.000105	1.27	A
6-Hydroxyhexanoic acid	422	$C_6H_{12}O_3$	25345715±3137799.56a	15581999±2678546b	6470339±1507588.97c	$8.79E^{-06}$	1.24	A
Prephenate	177.6	$C_{10}H_{10}O_6$	4304008±1284808.98a	1804134±54845.68b	403070.9±49497.11c	$4.21E^{-05}$	1.23	A
Propionylcarnitine	243.7	$C_{10}H_{19}NO_4$	29485.15±9143.35c	2926857±316100.6a	171072.1±22051.19b	$1.05E^{-09}$	1.20	A
N-Acetylmannosamine	100.3	$C_8H_{15}NO_6$	4120763±548885.66c	7455252±363816.3b	19314006±1230302.18 a	$6.36E^{-09}$	1.19	A

L-Carnitine	92.4	$C_7H_{15}NO_3$	4525598±1168509.36c	10598385±2715306a	5939935±2290437.86b	0.005869	1.15	A
Inosine	240.1	$C_{10}H_{12}N_4O_5$	19569.06±14923.37c	97827.9±61240.27b	745836.1±111054.99a	0.002868	1.10	A
Mannitol	128.5	$C_6H_{14}O_6$	353068.1±78381.11c	508315±270626.9b	961209±319409.8a	0.015069	1.07	A

Different letters indicate significant differences between groups ($p < 0.05$)

Table S4. Category B (exogenous-derived residues) detected in the samples.

name	Rt(s)	formula	*Lpb.plantarum* (means± SD)	*Lab.bulgaricus* (means± SD)	CK (means± SD)	**VIP**	*P*.value (FDR-adjust)	Category
4-Methylbenzaldehyde	227.9	C_8H_8O	108077.85 ± 8203.11c	853286.55 ± 66215.04a	701787.09 ± 76845.69b	1.8	$1.52e^{-10}$	B
1-Aminocyclopropanecarboxylic acid	108	$C_4H_7NO_2$	1146249±87917.28b	1433816±115709a	133635.4±9087.21c	1.8	$1.33e^{-11}$	B
Apigenin	211.8	$C_{15}H_{10}O_5$	75320.51 ± 12194.39b	135867.53 ± 17703.54a	104883.35 ± 13991.44a	1.79	0.0009	B
Eucalyptol	504.2	C10H18O	289677.56 ± 53822.09b	484946.02 ± 49076.54a	211831.05 ± 65964.19b	1.78	0.0329	B
N-Methyl-D-aspartic acid	127.6	$C_5H_9NO_4$	617888.26 ± 91323.88a	498955.33 ± 129429.01a	81121.52 ± 3106.95b	1.61	0.0042	B
Hippuric acid	389.8	$C_9H_9NO_3$	46507.77±47307.70c	49049.86±70445.41b	844844.8±115490.28a	1.63	0.000652	B
Palmitic acid	973.7	$C_{16}H_{32}O_2$	98138.61±17684.58c	174951.6±14241.06a	102493.4±5995.86b	1.55	0.000276	B
Melanin	78.7	Complex polymer	2244943±404987.7b	4871829±1662755a	2221002±430208b	1.50	0.0032	B
Catechol	843.1	$C_6H_6O_2$	1135834±161174.89c	1693941±154995.6b	8101441±601937.06a	1.48	$2.46E^{-09}$	B
Hydroquinone	448.2	$C_6H_6O_2$	2366568.08 ± 549280.45a	1549820.80 ± 437669.39a	2238847.94 ± 398514.93a	1.3	0.0528	B
Phenol	71.2	C_6H_6O	12482658.88 ± 4780858.35a	12146857.30 ± 2969906.61a	6626764.77 ± 1137152.83a	1.24	0.0174	B
Gallic acid	158.7	C7H6O5	30754.38±14093.37c	2166923±116797.1a	154465.4±23741.34b	1.16	$8.46E^{-08}$	B
Geranic acid	345	$C_{10}H_{16}O_2$	309834.75 ± 72873.13a	514291.35 ± 114532.68a	240249.37 ± 68302.90b	1.1	0.0065	B
gamma-Glutamylalanine	161.5	$C_7H_{11}N_2O_5R$	106388.46 ± 7479.11b	115884.28 ± 10927.89a	132989.45 ± 3723.70a	1.08	0.004	B
Linoleic acid	870.6	$C_{18}H_{32}O_2$	106329.2±54334.22c	148049.3±103723.3b	261298.8±29265.47a	1.07	0.031837	B
L-3-Phenyllactic acid	458.3	$C_9H_{10}O_3$	149414.35 ± 222956.09b	163043.93 ± 121320.99b	693828.67 ± 137378.08a	1.04	0.017	B
Caryophyllene epoxide	613	$C_{15}H_{24}O$	17700.04 ± 16004.38c	70095.42 ± 17229.45b	734774.93 ± 23439.32a	1.02	0.0001	B
Luteolin	563.6	$C_{15}H_{10}O_6$	32249.21 ± 14286.40a	13946.48 ± 6330.31a	4393.48 ± 2433.99b	1.02	0.0006	B
Glutaric acid	291.8	$C_5H_8O_4$	1619382.86 ± 230762.06a	1327154.08 ± 215397.11a	892094.36 ± 397338.83a	1.02	0.017	B
L-Arabinose	263.9	$C_5H_{10}O_5$	282013.37 ± 48957.52a	228918.32 ± 24837.64a	163661.47 ± 42093.73b	1.00	0.062	B

Different letters indicate significant differences between groups ($p < 0.05$).

Melanin has no single defined molecular formula because it is a complex, irregular polymer composed of various polyhydroxy and polyquinone units.

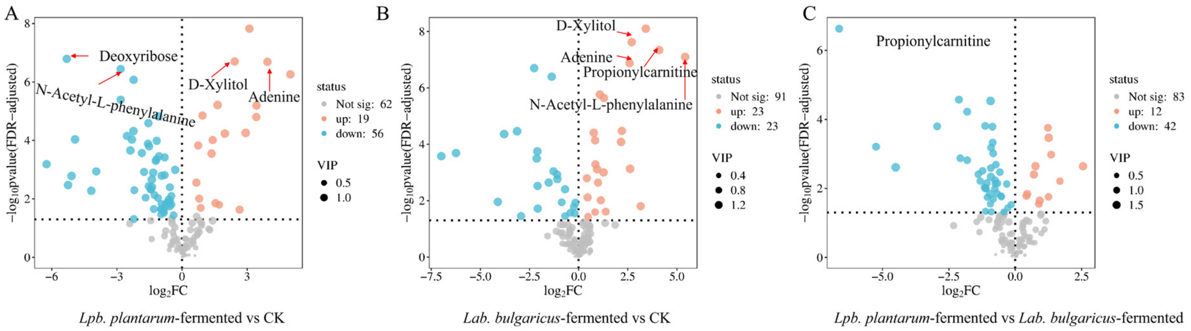

Figure S3. Volcano plots for *Lpb.plantarum* vs. control (CK) (A), *Lab.bulgaricus* vs. control (CK) (B), and *Lpb.plantarum* vs. *Lab.bulgaricus* groups (C).

Table S5. Volatile substances in control (CK), *Lpb.plantarum*-fermented and *Lab.bulgaricus*-fermented sample.

**Name**	**CAS#**	**RI**	**Rt(s)**	**Dt [RIPrel]**	Peak Volume			**VIP**
					CK	***Lpb.plantarum***	***Lab.bulgaricus***	
2,3-Pentanedione	C600146	1051.8	436.781	1.31688	39.696±3.275	1054.575±84.830	152.093±28.902	2.791
Butyl formate	C592847	994.6	371.707	1.51209	79.905±11.724	7960.759±239.399	4649.697±489.093	2.628
Ethyl acrylate-D	C140885	1053.6	439.018	1.40621	65.228±8.562	1262.554±459.573	72.133±20.141	2.328
(E)-2-Heptenal-D	C18829555	1329.1	878.496	1.67014	95.736±26.304	4142.704±202.638	263.945±141.180	2.234
10	*	1126.8	551.398	1.64222	51.534±0.530	1348.603±142.020	486.337±135.831	2.029
1-Hexanol-D	C111273	1368.8	956.435	1.64786	165.842±14.501	2776.077±31.940	140.993±24.998	1.999
1-Penten-3-one	C1629589	1024.6	404.489	1.30678	231.944±22.114	6353.724±112.844	2096.072±364.072	1.956
Hexanal	C66251	1084.7	479.274	1.55688	350.908±46.088	13894.360±206.932	6761.658±961.013	1.936
(E)-2-Pentenal-D	C1576870	1136.0	568.731	1.36088	326.711±35.201	6868.264±129.495	1147.060±197.817	1.848
15	*	1141.3	579.018	1.55254	19.899±2.970	296.632±6.941	68.353±9.763	1.830
Ethyl 2-methylbutanoate-D	C7452791	1050.1	434.677	1.64429	816.390±219.225	31.594±2.217	35.949±7.054	1.776
Propanal	C123386	785.1	234.235	1.15060	340.648±93.890	7216.542±62.168	4740.971±340.709	1.760
(E)-2-Octenal-M	C2548870	1437.4	1108.099	1.33554	231.979±5.871	3726.127±78.388	617.277±299.813	1.663
Dipropyl disulfide	C629196	1416.0	1058.430	1.26720	83.516±2.700	712.084±70.227	224.135±42.475	1.543
Ethyl propanoate	C105373	958.2	336.282	1.44785	274.986±68.617	709.124±271.835	74.471±18.575	1.517
1-Pentanol-D	C71410	1258.7	763.545	1.51459	214.022±18.006	1545.036±33.963	299.979±20.723	1.470
Acetic acid-D	C64197	1502.4	1273.858	1.16016	592.904±87.883	7023.553±426.527	10332.205±218.147	1.429

Ethyl isobutyrate	C97621	963.9	341.569	1.55491	2500.626±694.857	171.703±20.189	332.703±24.445	1.364
7	*	961.7	339.454	1.62671	801.751±47.548	67.260±1.833	96.434±4.000	1.356
(E)-2-Heptenal-M	C18829555	1328.6	877.534	1.25606	362.028±19.059	4255.461±44.918	1255.602±420.048	1.355
Ethyl acrylate-M	C140885	1053.9	439.385	1.13297	208.820±6.172	1861.786±139.086	596.814±112.015	1.337
Pentanal	C110623	984.3	361.132	1.43022	115.652±19.742	1413.783±35.734	599.038±68.371	1.334
2,3,5-Trimethylpyrazine	C14667551	1393.7	1008.962	1.15406	1970.764±1104.255	111.452±5.512	113.241±12.952	1.330
Ethyl 2-methylbutanoate-M	C7452791	1050.8	435.596	1.25145	1674.350±151.458	326.392±27.329	221.305±16.428	1.305
(E)-2-Hexenal-M	C6728263	1222.1	713.958	1.17993	156.817±7.153	1775.949±23.911	512.297±78.764	1.304
5	*	993.8	370.824	1.33257	145.904±13.038	869.760±13.087	1326.123±136.399	1.244
14	*	939.5	319.508	1.37970	31.516±3.865	403.727±30.223	55.822±11.498	1.214
2,5-Dimethylpyrazine	C123320	1351.3	921.366	1.11131	496.239±92.192	57.011±3.692	62.445±3.468	1.137
2-Pentylfuran	C3777693	1231.9	726.938	1.25145	612.623±21.729	2592.262±294.302	428.682±32.694	1.110
(E)-2-Hexenal-D	C6728263	1222.1	713.958	1.50859	451.135±15.113	3094.844±82.989	176.351±34.199	1.075
3-Methylbutanal	C590863	920.7	303.500	1.39495	190.054±24.129	1091.342±57.115	2536.618±73.365	1.074
9	*	956.5	334.695	1.58011	458.281±55.605	607.535±79.319	92.155±19.467	1.012
(E)-2-Octenal-D	C2548870	1437.4	1108.099	1.81803	154.512±21.030	671.921±51.201	134.142±14.668	1.012
(E,E)-2,4-Heptadienal	C4313035	1516.7	1313.364	1.20321	180.273±24.769	1295.051±46.259	385.812±93.929	1.009
1-Octen-3-ol	C3391864	1485.8	1229.318	1.16610	129.133±8.053	623.162±11.126	157.144±18.246	1.003
1-Penten-3-ol	C616251	1162.7	622.165	0.94117	422.874±13.419	2396.298±19.970	781.878±75.451	0.987
(E)-2-Butenal	C123739	1047.9	431.983	1.19720	822.860±42.568	2963.094±169.316	1563.431±61.669	0.977

(E)-2-Pentenal-M	C1576870	1136.0	568.731	1.10487	295.395±7.606	1826.179±17.950	960.560±104.905	0.969
(E)-2-Hexenol	C928950	1409.2	1043.010	1.34821	155.407±8.915	447.656±26.009	156.223±15.576	0.940
2-Propenal	C107028	848.2	263.845	1.06117	270.521±10.794	1004.236±9.798	387.523±26.507	0.937
1	*	1296.9	819.910	1.23230	6927.123±176.133	1019.159±30.300	290.421±9.890	0.920
2-Heptanone-D	C110430	1178.1	655.251	1.63148	286.972±30.196	1723.055±138.906	2132.506±11.763	0.901
Acetoin-D	C513860	1296.9	819.910	1.33156	31306.634±285.615	5107.625±157.610	768.524±61.775	0.892
Butyl propanoate-D	C590012	1141.8	579.888	1.71403	43.646±2.182	146.361±7.389	135.183±16.339	0.885
2-Ethylpyrazine	C13925003	1357.3	933.192	1.14920	132.124±58.080	128.873±22.571	55.371±5.038	0.846
1-Hexanol-M	C111273	1368.1	955.185	1.33156	1122.006±79.866	4760.395±14.243	829.338±91.802	0.843
2-Ethylfuran	C3208160	965.1	342.644	1.31408	661.574±5.262	129.153±3.756	537.320±12.398	0.823
2,3-Dimethylpyrazine	C5910894	1368.8	956.594	1.11441	419.782±54.325	90.643±4.994	70.023±15.837	0.740
Propyl butyrate-D	C105668	1174.7	647.899	1.68556	109.645±27.687	87.312±10.158	424.367±80.613	0.703
Nonanal	C124196	1400.9	1024.752	1.48445	195.720±26.644	778.908±13.342	371.662±40.525	0.685
4-Methyl-3-penten-2-one	C141797	1113.9	527.916	1.11154	142.492±19.434	395.376±3.843	182.766±6.256	0.650
12	*	1638.2	1704.402	1.15761	829.331±77.426	408.219±102.505	1189.233±39.842	0.642
Butanal	C123728	875.2	277.592	1.28033	310.624±3.736	958.202±30.558	419.062±26.589	0.636
2-Pentanone	C107879	979.5	356.406	1.36030	2311.877±184.123	4142.544±64.685	2960.556±36.316	0.621
2,4-Heptadienal	C5910850	1488.3	1235.783	1.21290	100.137±15.440	295.640±16.107	126.069±21.353	0.610
4	*	979.5	356.406	1.22427	3597.394±157.891	999.970±10.005	1574.973±88.073	0.603
1-Pentanol-M	C71410	1258.7	763.545	1.25556	1253.901±55.924	3009.998±52.149	1476.408±25.426	0.586

1-Octen-3-one-D	C4312996	1308.7	840.818	1.67624	80.042±15.352	183.877±16.671	65.170±10.940	0.578
2-Butanone-D	C78933	906.6	294.511	1.24633	1052.586±97.668	2892.027±22.466	2349.694±67.507	0.559
1-Heptanol	C111706	1487.7	1234.167	1.41138	120.579±19.897	189.416±5.138	130.926±24.292	0.554
13	*	1037.8	419.820	1.42695	222.399±1.747	683.993±11.587	324.024±26.836	0.552
2-Methyl-2-propanol	C75650	921.4	304.029	1.32316	705.930±78.495	1689.497±36.393	2114.724±22.183	0.535
3-Methyl-2-butenal-M	C107868	1200.3	686.054	1.09637	186.786±7.693	471.910±1.903	1492.832±19.879	0.519
Butyl propanoate-M	C590012	1142.2	580.807	1.28703	81.125±28.748	222.627±11.917	325.899±35.582	0.519
Acetic acid-M	C64197	1502.1	1273.034	1.06228	6270.299±334.496	15211.837±194.123	14948.491±178.089	0.508
Isoamyl acetate	C123922	1123.7	545.788	1.73947	62.592±20.443	23.342±1.679	22.792±2.588	0.500
Heptanal	C111717	1180.6	660.765	1.35535	249.431±5.249	595.578±13.277	337.594±17.260	0.486
2-Methyl-1-propanol-D	C78831	1095.5	496.254	1.36816	4029.070±48.149	1646.390±30.769	2076.264±37.020	0.477
1-Hydroxy-2-propanone	C116096	1310.1	843.466	1.21813	313.010±17.424	379.373±33.444	200.529±24.148	0.474
Propanoic acid	C79094	1637.8	1702.883	1.11607	327.535±10.778	740.921±5.744	1259.155±130.444	0.474
2-Methyl-1-propanol-M	C78831	1096.0	497.173	1.17032	2089.764±22.481	867.324±19.626	1476.588±92.682	0.471
Octanal-M	C124130	1294.7	815.952	1.41575	147.796±10.016	381.558±19.081	237.390±26.045	0.454
Benzaldehyde	C100527	1548.8	1407.107	1.15964	170.430±43.845	273.589±8.365	333.795±27.458	0.434
Dimethyl sulfide	C75183	763.1	224.718	0.95411	3617.589±275.160	2406.436±88.434	3639.256±94.892	0.425
2-Propanol-M	C67630	922.7	305.086	1.08888	585.252±14.328	294.977±7.124	232.089±6.864	0.403
2-Butylfuran	C4466244	1131.7	560.588	1.17886	338.491±26.889	170.018±22.452	164.699±5.837	0.401
2-Methylpropanoic acid	C79312	1692.9	1916.230	1.16771	407.521±18.107	599.834±38.413	764.674±14.126	0.398

1-Butanol-D	C71363	1148.3	592.755	1.38240	221.623±25.159	285.437±6.463	124.916±16.091	0.384
cis-2-Penten-1-ol	C1576950	1336.0	891.537	0.94566	282.973±4.147	609.325±8.987	560.371±25.257	0.377
11	*	1351.6	921.796	1.22635	490.333±44.312	472.338±8.305	161.930±17.745	0.320
2-Cyclohexen-1-one	C930687	1412.2	1049.913	1.12576	128.495±18.011	229.447±3.489	93.559±10.986	0.310
Acetoin-M	C513860	1296.9	819.910	1.07253	10032.435±165.601	5436.741±69.582	2592.011±136.098	0.289
2-Methylpropanal	C78842	801.6	241.638	1.27781	44.921±15.772	32.116±1.036	90.943±9.868	0.275
(E)-2-Nonenal	C18829566	1574.4	1486.304	1.41138	337.900±73.132	534.654±27.049	423.809±74.738	0.254
1-Propanol-M	C71238	1037.9	419.972	1.11054	1705.685±29.407	1316.961±10.238	1687.401±31.368	0.254
2-Heptanone-M	C110430	1180.6	660.765	1.26426	570.590±25.515	942.743±27.557	1371.979±23.637	0.244
8	*	954.2	332.581	1.69724	580.644±64.424	494.298±50.297	80.087±12.167	0.216
Ethyl acetate-D	C141786	886.2	283.408	1.33323	11704.324±380.070	7532.080±44.525	6400.087±118.332	0.208
Octanal-D	C124130	1295.2	816.978	1.81575	57.663±17.798	78.034±6.581	50.150±4.008	0.202
1-Octen-3-one-M	C4312996	1308.7	840.818	1.28411	937.678±22.381	794.979±19.989	392.408±52.138	0.201
2-Propanol-D	C67630	923.9	306.144	1.21862	1223.818±30.079	865.561±41.788	553.087±11.263	0.199
1-Butanol-M	C71363	1148.3	592.755	1.18313	797.036±35.837	619.170±22.751	469.943±2.416	0.188
3-Methyl-2-butenal-D	C107868	1202.6	688.953	1.36141	312.431±11.929	459.431±8.427	1687.915±9.987	0.185
1-Propanol-D	C71238	1038.7	420.892	1.25287	3861.202±64.823	3943.964±37.454	3222.187±20.229	0.164
Ethanol	C64175	932.4	313.362	1.13474	41277.298±1035.429	29818.760±32.319	35118.448±571.772	0.160
2-Isobutyl-3-methoxypyrazine	C24683009	1563.1	1450.746	1.23710	198.227±15.162	270.328±24.028	193.035±13.611	0.159
2-Undecanone	C112129	1696.0	1929.160	1.55339	627.124±86.895	821.753±56.124	591.189±62.789	0.158

Propyl butyrate-M	C105668	1174.3	646.980	1.26141	489.279±81.873	594.219±27.712	1011.761±54.639	0.145
Butyl acetate	C123864	1076.1	467.763	1.61297	36.194±11.024	35.803±4.411	285.503±44.879	0.136
3-Nonanone	C925780	1345.2	909.355	1.38057	376.135±92.283	456.842±36.644	188.831±19.534	0.133
3-Methyl-1-butanol-D	C123513	1210.1	698.447	1.49199	3462.130±125.128	3781.188±31.553	1223.080±25.406	0.132
2-Ethyl-1-hexanol	C104767	1539.0	1377.802	1.42884	339.162±35.521	273.396±29.230	224.505±27.677	0.121
2	*	1148.3	592.755	1.26568	528.166±22.393	419.761±5.048	259.033±16.398	0.119
3-Methyl-1-butanol-M	C123513	1210.1	698.447	1.24433	3197.360±15.882	2751.972±22.970	2130.462±8.184	0.113
Isobutyl acetate	C110190	1008.0	385.967	1.23437	659.894±2.527	750.956±9.289	534.715±11.219	0.110
1-Octanol	C111875	1653.9	1762.685	1.48077	679.905±127.701	664.784±22.817	673.372±62.343	0.075
Acetone	C67641	815.4	247.982	1.11407	15369.728±102.532	17464.385±71.153	15154.382±30.463	0.066
3	*	1013.8	392.401	1.09488	1126.061±12.597	1091.815±11.231	809.258±9.275	0.054
2-Butanone-M	C78933	907.5	295.040	1.05991	536.861±19.927	589.960±10.385	569.827±2.831	0.050
6	*	955.2	333.470	1.40653	1065.340±61.196	1046.806±8.950	418.924±23.506	0.048
Ethyl acetate-M	C141786	885.2	282.879	1.09770	1178.465±102.314	1184.722±26.169	1711.483±33.535	0.033
Acetaldehyde	C75070	723.0	208.327	1.02590	1721.528±93.825	1700.212±25.865	863.150±9.865	0.029
